# Mutation of Brain Aromatase Impairs Behavior and Neuroplasticity in Adult Zebrafish

**DOI:** 10.1111/jnc.70202

**Published:** 2025-08-25

**Authors:** Cassandra Malleret, Mélanie Blanc‐Legendre, Laëtitia Guillot, Harmony Lautrette‐Quinveros, Pavlina Pavlidi, Christina Dalla, Nikos Kokras, François Brion, Maryne Toupin, Frédéric Chalmel, Xavier Cousin, Thierry Dominique Charlier, Elisabeth Pellegrini

**Affiliations:** ^1^ Inserm, EHESP, Irset (Institut de Recherche en Santé, Environnement et Travail), UMR_S 1085 Univ Rennes Rennes France; ^2^ MARBEC, CNRS, Ifremer, IRD, INRAE Univ Montpellier Palavas‐les‐Flots France; ^3^ Department of Pharmacology, Medical School National and Kapodistrian University of Athens Athens Greece; ^4^ Second Department of Obstetrics – Gynecology, Aretaieio Hospital, School of Medicine National and Kapodistrian University of Athens Athens Greece; ^5^ First Department of Psychiatry, Medical School National and Kapodistrian University of Athens Athens Greece; ^6^ Unité Écotoxicologie In Vitro et In Vivo, UMR‐I 02‐SEBIO, Parc ALATA Institut National de l'Environnement Industriel et des Risques (INERIS) Verneuil‐en‐Halatte France; ^7^ ImPACcell Platform, Biosit University of Rennes Rennes France

**Keywords:** *Cyp19a1b* gene, dopamine, estrogens, serotonin, teleost fish

## Abstract

Brain aromatase, an enzyme responsible for the local synthesis of estrogens, plays a key role in regulating behavior and neuroplasticity in mammals. In teleost fish, brain aromatase is encoded by the *cyp19a1b* gene, which is strongly expressed in radial glial cells; however, the specific functions of this enzyme are currently unknown. To investigate its role, a *cyp19a1b*‐mutant zebrafish line was generated using gene‐editing techniques. Behavioral, neurogenic, and neurotransmission‐related parameters were assessed in adult male and female zebrafish. Behavioral analysis highlighted significant alterations in mutant zebrafish, including changes in swimming activity, boldness, sociability, and aggression, with a stronger effect in males compared to females. Beyond these behavioral modifications, mutant zebrafish exhibited disrupted cell proliferation patterns, as assessed by PCNA immunofluorescence in key forebrain regions. Specifically, proliferation decreased in the telencephalon and in the caudal hypothalamus of mutant zebrafish while increasing in the olfactory bulbs. The number of dopaminergic and serotonergic neurons, visualized by immunofluorescence, remained unchanged. Similarly, HPLC‐ED quantification of monoamines and their metabolites showed no significant differences between mutant and wild‐type zebrafish. To further explore the impact of the *cyp19a1b* mutation on gene expression, transcriptomic analysis was performed using BRB‐Seq technology. Gene expression analyses identified several processes affected by the mutation, including cell proliferation, apoptosis, estrogen signaling, neuroplasticity, and behavioral regulation, in a sex‐ and region‐dependent manner. In conclusion, our results demonstrate that several behaviors, including locomotor activity, sociability, aggressiveness, and anxiety, exhibit marked sexual dimorphism. They show that the *cyp19a1b* mutation affects locomotor activity in a context‐dependent manner, increases boldness, and reduces aggressiveness. In addition, transcriptomic analyses revealed widespread dysregulation of gene expression, which likely contributes to the observed behavioral alterations. Taken together, these findings underscore the crucial role of brain aromatase in the neurobiological regulation of diverse behaviors.

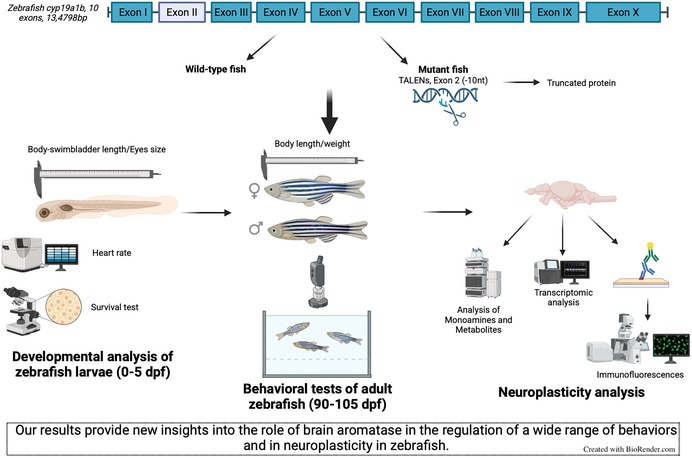

## Graphical abstract

This study investigates the functional role of brain aromatase, encoded by the *cyp19a1b* gene, in zebrafish neurodevelopment and adult behavior. Using TALENs technology, we introduced a 10‐nucleotide deletion in exon 2, generating a zebrafish line that expresses a truncated aromatase protein. Early developmental parameters, including heart rate, survival, eye size, body, and swim bladder lengths, were assessed. In adults, we conducted behavioral tests and analyzed neuroplasticity through immunofluorescence, transcriptomic profiling, and quantification of monoamines and their metabolites. Our findings provide new insights into the role of brain aromatase in regulating a wide range of behaviors and neural plasticity mechanisms in zebrafish.

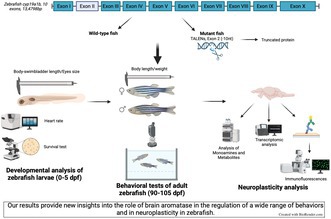

**Abbreviations**
ArKO: aromatase knockout
AroB: aromatase B
ATD: 1,4,6‐androstatriene‐3,17‐dione
D: Dorsal telencephalic area
Di: lateral zone of D
Dm: medial zone of D
FBN‐ARO‐KO: forebrain‐neuron‐specific aromatase knockout
Hc: caudal zone of periventricular hypothalamus
Hd: dorsal zone of periventricular hypothalamus
Hv: ventral zone of periventricular hypothalamus
OB: olfactory bulb
PGZ: periventricular gray zone of optic tectum
PPa: parvocellular preoptic nucleus, anterior part
PTN: posterior tuberal nucleus
PVO: paraventricular organ
RGCs: radial glial progenitor cells
RRID: research resource identifier (see https://rrid.site)
Tel: telencephalon
Vv: ventral nucleus of ventral telencephalic area

## 1 Introduction

Aromatase, also known as cytochrome P450 aromatase, is a key steroidogenic enzyme involved in estrogen biosynthesis by converting aromatizable C19 androgens into C18 estrogens. Expressed in gonads and in many organs, including the brain, aromatase plays a crucial role in numerous physiological and behavioral functions (Aspesi and Cornil 2024; Brann et al. 2022). In mammals, 17β‐estradiol (E2), one of the main products of aromatase, regulates neurogenesis and neuroplasticity during development and adulthood. A wealth of data has pointed to its role in many cellular processes such as proliferation of neural progenitor cells, survival, migration, and differentiation (Azcoitia et al. 2018; Diotel et al. 2018). E2 also modulates many neurotransmitter systems through regulatory effects on synthesis, release, and signaling (Bendis et al. 2024). The key role of aromatase in neurogenic processes has been highlighted in aromatase knockout (ArKO) mice exhibiting more apoptotic neurons in the cerebral cortex, fewer proliferating progenitor cells in the hippocampus, and fewer surviving cells in olfactory bulbs (Brus et al. 2016; Hill et al. 2009). ArKO mice also display strong deficits in both olfactory investigations of social signals and sexual behavior (Brus et al. 2016; Pierman et al. 2006). In the more recent FBN‐ARO‐KO (forebrain‐neuron‐specific aromatase knockout) mouse model, the specific deletion of aromatase in forebrain neurons leads to a significant decrease in synapse density, emphasizing the crucial role of neuron‐derived estradiol in synaptic plasticity (Lu et al. 2019). Moreover, this model revealed significant impairments in hippocampal‐dependent spatial reference learning and memory (Lu et al. 2019), suggesting that brain aromatase may play key roles in cognitive behaviors in mammals.

Aromatase protein is abundant in the teleost brain, where its distribution is much broader than in birds and mammals. As a result, overall brain enzymatic activity is 100–1000 times higher than in other vertebrates (Callard et al. 1990). Teleost fish are unique in that they have two aromatase‐encoding genes arising from a genome duplication event approximately 320–350 million years ago (Taylor et al. 2001): one gene, *cyp19a1b*, is predominantly expressed in the brain and encodes aromatase B (AroB), while the other, *cyp19a1a*, is primarily expressed in the gonad. Several studies have shown that the *cyp19a1b* gene is expressed in radial glial progenitor cells (RGCs) bordering the ventricles (Forlano et al. 2001; Menuet et al. 2005). These RGCs exhibit mitotic activity and divide to generate newborn cells, which then migrate away from the ventricles and, in some cases, differentiate into neurons (Pellegrini et al. 2007; Pérez et al. 2013). The functional significance of AroB expression in progenitor cells is currently unknown.

Given that estrogen receptors are expressed in the periventricular regions that contain radial glial cells, it is hypothesized that aromatase B, through its role in local production of estradiol, modulates cell proliferation, neuronal differentiation, and neuroplasticity (Menuet et al. 2002; Coumailleau et al. 2015; Diotel et al. 2020).

Additionally, the proximity of AroB‐positive radial glial extensions to dopamine, serotonin, and vasopressin neurons strongly suggests that locally produced estradiol could also modulate neurotransmission (Pérez et al. 2013; Shaw, Lu, et al. 2023; Xing et al. 2015). Much of our understanding of the function of aromatase in teleosts has been shaped by studies using pharmacological aromatase inhibitors. Inhibition of aromatase activity with 1,4,6‐androstatriene‐3,17‐dione (ATD) has been shown to impair brain cell proliferation (Diotel et al. 2013; Lin et al. 2016). Treatment with the aromatase inhibitor fadrozole disrupted sex differentiation and reduced aggressiveness (Huffman et al. 2013; Jalabert et al. 2015; Kitano et al. 2000; Kwon et al. 2000; Shaw 2018; Zubizarreta et al. 2020). Since chemical aromatase inhibitors are not selective for the two forms of aromatase, these studies are limited in their ability to distinguish the specific contributions of brain‐derived versus gonadal‐derived aromatase/estradiol to these effects. However, even though a recent study, based on the generation of a *cypa19a1b* mutant zebrafish line, highlighted its role in sexual behavior in females (Shaw, Therrien, et al. 2023), our understanding of the role of AroB in the brain is far from clear.

To investigate the functional link between brain aromatase, behavior, and underlying neuronal mechanisms, we used zebrafish, a well‐established vertebrate model that shares fundamental neurogenesis mechanisms with other vertebrates. Moreover, in contrast to mammals, where brain aromatase expression is more limited and restricted to specific neuronal populations, zebrafish display exceptionally high levels of brain aromatase (*cyp19a1b*) expression, specifically in radial glial progenitor cells, as mentioned above. These features make zebrafish particularly well suited to investigate the impact of local synthesis of estrogens in the brain on neural development, plasticity, and function.

To explore the mechanisms underlying the role of *cyp19a1b*, we first generated a zebrafish mutant line. We carefully characterized the model and investigated how the *cyp19a1b* mutation influences a range of biological functions. We focused on both reproductive and non‐reproductive behaviors, aiming to capture the broader impact of this mutation on the zebrafish's overall behavioral repertoire. Beyond behavior and reproduction, we turned our attention to the brain and performed transcriptomic analyses to assess changes in gene expression in mutant zebrafish. We also investigated neurogenesis, with a particular focus on brain cell proliferation. Finally, we explored monoaminergic neurotransmission, with a specific focus on the dopaminergic and serotonergic systems, given their known involvement in motivation and behavior.

## 2 Materials and Methods

### 2.1 Fish Housing

Zebrafish were handled and euthanized in agreement with the guidelines for the use and care of laboratory animals and in compliance with French and European regulations on animal welfare. The experiments were carried out on wild‐type (WT) AB strain (RRID: ZIRC_ZL1) and *cyp19a1b*‐mutant zebrafish ( *Danio rerio* ) lines. Fish were kept at the MARBEC Palavas Experimental Marine Platform (agreement D34121926 for animal experiments; behavioral and adult immunostaining analyses, embryonic development, and survival study) and at Biosit Rennes (agreement C35‐238‐40; embryonic survival study, sex‐ratio analyses, aromatase activity and monoamine assays, and transcriptomic study).

The zebrafish were maintained under standard housing conditions with a photoperiod of 14:10 h light: dark. The water temperature was kept constant at 28°C ± 1°C, with a pH of 7.0–7.5. Fish density was maintained at approximately 8 fish per liter in a recirculating aquarium system. Fish were fed twice daily with commercial dry pellets. The welfare of zebrafish was monitored daily.

The sample size was determined based on our expertise, previously published studies, and other research conducted under comparable conditions, with relevant references provided in the sections below.

### 2.2 Cyp19a1b‐Mutant Line and Genotyping

A *cyp19a1b*‐mutant zebrafish line was generated by the TALENs technology (Amagen, France). The mutant line does not exhibit any harmful phenotypes, and zebrafish from this line were euthanized in accordance with French and European regulations. Genotyping was performed on tail samples collected after the animals were euthanized with 500 mg/L tricaine solution (MS‐222). Genome information for zebrafish *cyp19a1b* was obtained from the National Center for Biotechnology Information (https://www.ncbi.nlm.nih.gov/gene/60640). The TALENs were generated with left and right arms that target the second exon of *cyp19a1b* to obtain a 10‐nucleotide deletion and induce a frameshift. It also introduces a TauI restriction site, enabling mutated fish to be identified by PCR and restriction genotyping. Initial genotyping was carried out by Sanger sequencing by Eurofins (Nantes, France) and allowed for validation of the PCR genotyping approach. This latest method was performed on a 1 mm diameter punch from the caudal tail, lysed in 200 μL of 50 mM NaOH at 95°C for 45 min, and the reaction was stopped by adding 20 μL of 1 M Tris (pH 8). Then, we centrifuged at 11,000 g for 5 min, and we amplified a fragment of genomic AroB flanking the site of deletion using the following primers (Zf_AroB_Forward 5′GCAGATTTTCAGCCTCATTTCC3′; Zf_AroB‐Reverse 5′GATCCAAACTCGAGCGATGC3′). PCR was performed using the HotStarTaq DNA polymerase (Qiagen, cat. no. 203203). The DNA PCR was also sent for sequencing. A digestion step was performed at 55°C for 2 h with the TauI restriction enzyme (ThermoFisher Scientific, USA, cat. no. 10568170), which cuts through the GCSG^C site to identify wild‐type individuals from heterozygous and homozygous mutants.

### 2.3 Embryonic Survival

For the survival test, fertilized eggs were collected and distributed by 100 in glass crystallizers filled with E3 embryo buffer (composition); then maintained in an incubator at 28°C ± 1°C. Survival was counted by microscopic observation every 24 hpf until 120 hpf. This was performed on 40 (WT) and 44 (mutant) egg‐layings.

### 2.4 Development and Rearing

In the evening before the test, pairs of adult zebrafish were set in 1 L spawning boxes. In the morning, fertilized eggs were collected and assessed for quality. Only homogeneous spawns with eggs ≤ 4 hpf were kept. Mixtures of approximately 30 eggs from 3 to 5 different spawns were kept in glass crystallizers filled with ISO 7346‐2 water (1996) (294 mg/L CaCl_2_·2H_2_O, 123.3 mg/L MgSO_4_·7H_2_O, 63 mg/L NaHCO_3_, 5.5 mg/L KCl, pH 7.8); then incubated at 27°C ± 1°C. Embryonic development was monitored daily by microscopic observation.

For rearing, larvae were transferred to 1 L aquariums (approximately 30 larvae per aquarium) at 5 days post‐fertilization (dpf). At approximately 30 dpf, inserts were removed and fish were kept in 10 L tanks until the end of the experiment. This was performed in triplicate.

### 2.5 Heart Rate Measurement

Heartbeat frequency was recorded in 48 hpf embryos. Two hours before measurement, embryos were individually transferred to 24‐well plates containing 1 mL of ISO water. They were gently positioned with the heart facing upwards. Then, 30 s videos were taken under a microscope (×40) using the ICCapture Software. Video files were analyzed using DanioScope (Noldus) to extract heartbeat frequency as beats per sec and beats per min. This experiment was conducted on embryos from three different spawns (total *n* = 25 WT and *n* = 35 mutants).

### 2.6 Total Length, Swim Bladder, and Eye Size

Total body length, eye size, and swim bladder size were measured in 120 hpf larvae. From fertilization onwards, embryos were reared under standard conditions as described above. On day 5, larvae were individually transferred to 24‐well plates (1 larva per well) in 2 mL of ISO water. They were then anesthetized using 50 mg/L benzocaine (CAS nr 94‐09‐7) for 2 min. Then, 1.5 mL of medium was removed to ease positioning of the larvae for imaging. Pictures were taken under a binocular microscope (×20) using ICCapture Software. Finally, larval body length, eye size, and swim bladder size were determined using the image analysis software DanioScope (Noldus). This experiment was carried out in duplicate (*n* = 25 WT and *n* = 21 mutants).

### 2.7 Behavior

Behavioral tests were carried out on adults (90–105 dpf) in a dedicated room with controlled temperature and photoperiod synchronized with the rearing room. Different behavioral traits were sequentially evaluated. The whole sequence of tests was performed in three sessions during three successive weeks using naive fish of each genotype and sex for each session. Tests are described in detail below, while only the outline is presented here. Each session started with a shoaling behavior test in the morning of day 1, which is a group test performed with groups of 6 fish. After each shoaling behavior test, some individual fish (females and males) were randomly and evenly taken from each “shoaling groups” and transferred to annotated 1 L tanks for further behavioral tests. A spatial memory test was then performed in the afternoon of day 1. Associative learning capacity by classical conditioning was evaluated on day 2 (whole day), exploration capacities were evaluated on day 3, and then anxiety and sociability were assessed in the morning and afternoon of day 4, respectively. Aggressiveness was evaluated on day 5. The sequence and order of tests were dictated by practical reasons, including the duration of each test. Behavioral tests were not performed blind to the sex nor genotype.

Over the three sessions, a total of 13 WT and 11 mutant groups of 6 fish were tested for a total of 39 females and 39 males in WT and 33 females and males for mutants. As described above, some individuals (females and males) were randomly taken from each “shoaling groups”, resulting in a total of 50 individual fish (*n* = 12 WT females, 13 WT males, 13 mutant females, and 12 mutant males). These fish were then submitted to all individual tests following the order indicated above.

Fish were isolated in individual 1 L tanks in visual contact between neighbor tanks over the week of testing. Water was renewed daily, and fish were fed once a day in the evening with freshly hatched artemia. Genotypes and sexes were randomized to avoid bias due to the time of day for testing. For all tests, fish behavior was camera‐recorded, and individuals were automatically tracked using Ethovision software (Noldus). Water in behavioral devices was changed between every run except when mentioned otherwise in the detailed section below. Within the 5 min following the last behavioral test (aggressiveness test), fish were euthanized using 500 mg/L benzocaïne solution, measured, and weighed to evaluate possible differences in growth between WT and mutants. Heads were sectioned at the top of the spinal cord and transferred to 4% paraformaldehyde (PFA) in 0.01 M phosphate buffered saline (PBS) solution for immunostaining analyses. All behavioral analyses were performed on three distinct batches for a total of *n* = 50 individuals.

#### 2.7.1 Shoaling

Groups made of 6 fish (3 males and 3 females from the same genotype) were transferred to a narrow 3 L tank to evaluate the cohesion of the shoal (Pham et al. 2012). Their behavior was camera‐recorded (lateral view, DMK33GP1300e, The Imaging Source) for 6 min, including 3 min for acclimatization and 3 min used for video analysis. Water was exchanged between each group, and a total of 13 (WT) and 11 (Mutants) groups were tested. Data were analyzed as individual distance traveled (mm) and average interindividual distance (mm).

#### 2.7.2 Spatial Memory

The y‐maze (arm size 25 cm * 8 cm * 15 cm (Length * width * hight)) consists of three arms, each visually identifiable by fish thanks to different symbols. During the first 5 min session, the fish was allowed to navigate between two arms, with no access to the third arm. After a 60 min interval, the fish was submitted to a second 5 min session with access to all three arms. As a marker for short‐term spatial memory, the time spent in the “novel” arm is supposed to increase (Cognato et al. 2012). The behavior of fish was camera‐recorded (top view, DMK33GP1300e, The Imaging Source). Data were analyzed as distance traveled (mm), number of alternations between arms, and time spent in the novel arm (s).

#### 2.7.3 Associative Learning

We evaluated the learning capacity of zebrafish using classical conditioning performed according to Zantiks protocol and the Zantiks AD unit; with minor modifications (Brock et al. 2017; Valente et al. 2012). Fish were exposed to an unconditioned aversive stimulus (US, mild electric shock) associated with a conditioned visual stimulus (CS, visual cue checkerboard pattern) that fish were conditioned to avoid. Visual cues were presented on the integrated screen below the testing tank, and aversive stimuli were given by metal plates inserted at both ends of the testing tank. One session was divided into an acclimatization period to ensure the absence of pattern preference (40 min), followed by the conditioning period of 9 US/CS associations (90 s total). Finally, a last 1 min probe period was used to evaluate the preference of the fish to spend time outside the checkerboard pattern. Data were analyzed as time spent outside the checkerboard area (s), number of visits to the checkerboard area, and distance traveled (mm).

#### 2.7.4 Exploratory Capacities

Exploration propensity was evaluated using a Z‐shaped maze of 45 cm * 75 cm * 7.5 cm (L * l * h), divided into 4 corridors and 16 virtual zones (Chapman et al. 2010; Vignet et al. 2014). The fish was transferred to a shelter area initially isolated from the rest of the labyrinth by a closed door and covered by an opaque plate (acclimatization phase). After 2 min, the door was opened and the behavior of the fish was recorded (top view, DMK33GP1300e, The Imaging Source) for 5 min from the time it exited the shelter area. Water was thoroughly aerated and mixed between each run and totally renewed every 5 runs. Data were analyzed as time to exit the shelter area (s), distance moved (mm), time spent in the proximal zone (s), time spent in the distal zone (s), time to reach the distal zone (s), time to reach the furthest area (zone 16, s), and time spent in zone 16 (s).

#### 2.7.5 Anxiety and Sociability

These two tests were performed successively using the same narrow 3 L tank filled with 1.6 L of water. Dark gray walls lined the two ends of the tank. On one side, the wall was removable to allow the view of 4 congeners (2 males and 2 females) behind a transparent Plexiglas wall.

The novel tank diving test was carried out first to evaluate the anxiety level of fish by looking at their vertical positioning (Levin et al. 2007). A fish was transferred into the tank with the removable wall lowered, and its behavior was recorded (lateral view, DMK33GP1300e, The Imaging Source) for 5 min. Data were analyzed as distance traveled (mm), time spent in the virtual top zone (s), and number of visits to the upper zone.

Two minutes after the end of the novel tank test, the wall was raised to allow visual contact with the four congeners. The behavior of the fish was recorded during 5 min (lateral view, DMK33GP1300e, The Imaging Source). Data were analyzed as distance traveled (mm), number of visits to the social area (i.e., next to congeners), and time spent in the social area (s).

#### 2.7.6 Aggressiveness

An individual was transferred into a narrow 3 L tank where a mirror covers one extremity. During the first period of 2 min of acclimatization, the mirror was hidden behind an opaque door. Then, the door was automatically lifted, and the behavior of the fish was recorded for 5 min (lateral view, DMK33GP1300e, The Imaging Source). Data were analyzed as distance traveled (mm), number of visits to the mirror zone, and time spent in the mirror zone (s).

### 2.8 Reproduction, Reproductive Behavior, and Sex Ratio

The sex ratio was determined on wild‐type and mutant zebrafish that were raised to sexual maturity (4 months) after mass egg‐laying (1 female for 3 males). The number of males and females in three populations was counted after gonad analysis by dissection.

The test took place in a dedicated room with controlled temperature and photoperiod synchronized with the rearing room. The afternoon before the test started, one male and one female from the same genotype were placed in a 1 L spawning box divided into two equal sections by a grid insert to separate individuals. A spawning area consisting of a circular insert (45 mm diameter) filled with glass marbles was placed in one corner of the spawning box on the female side. A maximum of nine couples were tested simultaneously (for a total of 22 WT and 24 mutants). In the morning of the test, at light onset, dividers were removed and the behavior of fish was recorded laterally for 3 h (DMK33GP1300e, The Imaging Source). At the end of the recording, fish were transferred back to the rearing system and spawns were collected. Behavior was evaluated as total distance traveled (cm), time spent in the spawning area (s), body contact time (s), and average interindividual distance (cm). In addition, spawning successes (the number of successes/number of attempts per genotype), number of eggs, and fertilization rate were systematically quantified. Additional spawns were collected from paired reproduction events in the same conditions (without camera recording) for a total of 35 WT and 37 mutants.

### 2.9 Sampling and Brain Sections

After behavior recording, the animals were euthanized within 5 min using 500 mg/L benzocaine solution. Total length and body weight were taken to evaluate possible differences in growth between WT and mutants. The heads were cut and immersed for 2 days at 4°C in 4% PFA in 0.01 M PBS (pH 7.4). The samples were washed in PBS, then immersed in ethylene diamine tetraacetic acid for decalcification (EDTA 0.5 M pH 7.5) for 2 weeks. Cryoprotection was performed in PBS solutions containing sucrose, at 15% for 24 h and then at 30% for 48 h, at 4°C. Finally, heads were included in a cup filled with a mounting medium (NEG‐50, ThermoFisher Scientific, USA), oriented under a binocular loupe, and frozen into isopentane at −60°C. Once frozen, the samples were stored at −80°C until further processing for analysis of the impact on neuroplasticity. 20 μm‐thick coronal sections were then obtained using the cryostat Microm HM 560 (ThermoFisher Scientific, Lithuania) and mounted on Epredia SuperFrost Plus Adhesion Microscope Slides 25 × 75 × 1 mm (ThermoFisher Scientific, USA). The sections were placed on three slides successively to obtain three replicates per brain. They were then dried for at least 30 min on a RaymondALamb slide dryer, then stored at −20°C until immunostaining.

### 2.10 Immunofluorescence

The dilution of the primary and secondary antibodies used in this study is summarized in Table S1. The AroB antibody was raised against the synthetic peptide CNSNGETADNRTSKE corresponding to the last 15 residues of the zebrafish AroB sequence (AY780257.1; Menuet et al. 2005; Pellegrini et al. 2007). PCNA (Proliferative Cell Nuclear Antigen) antibody (cat. no. MO879) was used to label cells in S phase at the time of euthanasia (Pellegrini et al. 2007). For identification of dopaminergic neurons, we used an anti‐tyrosine hydroxylase (TH) (RRID: AB_390204) previously described (Godoy et al. 2020). Serotonergic neurons were visualized with a rabbit anti‐5HT antibody provided by Dr. Yves Tillet (Pérez et al. 2013; Tillet 1987).

An antigen retrieval was carried out in a sodium citrate buffer (0.1 M, pH 6) at 80°C for 30 min. Nonspecific binding was blocked for 45 min at room temperature in PBS containing 0.2% Triton 1× and 0.5% milk powder. Sections were then incubated with mouse anti‐PCNA antibody, combined with another primary antibody (Table S1). This primary antibody cocktail was applied to sections with PBS 0.01 M – 0.5% milk, and slides were kept overnight at room temperature (RT) in a humidified dark chamber. After several washes, tissue sections were incubated with the appropriate secondary antibody (RRID: AB_2534091 and AB_2576217 for PCNA and TH/5‐HT, respectively) for 1 h 30 at RT in the same dark chamber. Then, slides were rinsed in PBS 0.01 M and in PBS with 0.1% Triton 1×. Slides were mounted with Vectashield Vibrance Antifade with DAPI (cat. no. H‐1800‐10, Eurobio Scientific) to allow visualization of cell nuclei.

### 2.11 Immunofluorescence Staining Analysis

Neuroanatomical structures were identified with DAPI staining. The nomenclature for brain nuclei is according to that established in the zebrafish atlas “Neuroanatomy of the Zebrafish Brain: A Topological Atlas” (Wullimann et al. 1996). Proliferative and neurotransmission activity was assessed by quantifying the number of PCNA, TH, and 5HT‐labeled cells on several 20 μm‐thick brain sections (Male: *n* = 8 WT and 9 mutants; Female: *n* = 7 WT and 8 mutants). All individuals were processed and analyzed in a blinded procedure, from inclusion to cell quantification, as experimental group assignment was carried out by an independent person. The mean number of PCNA cells was calculated on two consecutive sections of olfactory bulbs (OB), dorsal telencephalic area (D), parvocellular preoptic nucleus, anterior part (PPa), ventral zone of periventricular hypothalamus (Hv), dorsal zone of periventricular hypothalamus (Hd) and caudal zone of periventricular hypothalamus (Hc); three consecutives sections for Ventral nucleus of ventral telencephalic area (Vv) corresponding to the subpallium, Medial zone of D (Dm), corresponding to the amygdala, Lateral zone of D (Di), corresponding to the hippocampus, thalamus and pituitary. Dopaminergic neurons were counted on two consecutive sections of OB, PPa, posterior tuberal nucleus (PTN), and Hc; three consecutive sections for ventral nucleus of ventral telencephalic area (Vv) and thalamus. The density of serotonergic neurons was too high to set apart individual neurons. Quantification of serotonergic neurons was thus performed indirectly by the measure of immunofluorescence intensity using the FIJI software (Schindelin et al. 2012). Following calibration, images were transformed into 8‐bit images and optical density (OD) in a surface of interest (Area) was measured and averaged in two consecutives sections of paraventricular organ (PVO), PTN and Hc. For 5‐HT immunofluorescence, brain sections were scanned using Nanozoomer 2.0‐RS, Hamamatsu Photonics, K. K. (Platform H2P2, Rennes, France) at 20× resolution. Images were acquired with NDPScan 3.3 software and visualized with NDP.view2 (2.9.29, Hamamatsu Photonics K.K.). For PCNA and TH quantification, sections were analyzed with an epifluorescence microscope Zeiss Imager.Z1 equipped with an AxioCam MRm camera (Zeiss, Germany). Slides were observed with a 20X lens in AxioVs40 software.

### 2.12 Analysis of Monoamines and Metabolites

High‐performance liquid chromatography with electrochemical detection (HPLC‐ED) was performed on whole brain samples (*n* = 9 WT females, 10 WT males, 9 mutant females, and 10 mutant males). Brains were homogenized and deproteinized in 0.1 N perchloric acid solution containing 7.9 mM Na_2_S_2_O_5_, 1.3 mM EDTA, and 70% HClO_4_, and sonicated for 45 s with a 5 s on and 5 s off cycle. Samples were centrifuged at 20,000 *g* for 30 min at 4°C. Protein quantification was performed on the pellet by the Compat‐Able BCA Protein Assay Kit (cat. no. 23229, Thermo Fisher Scientific, Rockford, USA) following the manufacturer's instructions, and the supernatant was stored at −80°C until analysis. Analytical measurements were performed in a blinded procedure using an LKB2248 (Pharmacia LKB Biotechnology, Uppsala, Sweden) HPLC pump coupled with a BAS LC4C (Bioanalytical Systems, West Lafayette, IN, USA) electrochemical detector as previously described (Kokras et al. 2018), with some minor modifications. The working electrode was glassy carbon, the reference electrode was Ag/AgCl, and the column used was Aquasil C18, 150 mm × 2.1 mm, 5 μm (Thermo Fisher Scientific, Massachusetts, USA). The voltage of the working electrode was set at +800 mV. In all samples, reverse phase ion pair chromatography was used to assay norepinephrine (NE), serotonin (5‐HT) and its metabolite 5‐hydroxyindoleatic (5‐HIAA), as well as dopamine (DA) and its metabolites 3,4‐dihydroxyphenylacetate (DOPAC), homovanillic acid (HVA), and 3‐methoxytyramine (3‐MT). The mobile phase consisted of a 50 mM phosphate buffer regulated at pH 3.0, containing 300 mg/L octyl sulfate sodium salt (MP Biomedicals LLC, Irvine, CA, USA) as the ion pair agent and 20 mg/L Na_2_EDTA; acetonitrile (Merck, Darmstadt, Germany) was added at 8%–10% v/v concentration. The sensitivity of the assay was tested for each series of samples using external standards, which were prepared daily in 0.2 N perchloric acid solution containing 7.9 mM Na_2_S_2_O_5_ and 1.3 mM Na_2_EDTA. Samples were quantified by comparison of the area under the curve (AUC) against these reference external standards using PC‐compatible HPLC software (Clarity, Data‐Apex, Czech Republic). The 5‐HT and DA turnover rates (5‐HIAA/5‐HT, HVA/DA, DOPAC/DA, and 3MT/DA) were calculated as indices of serotonergic and dopaminergic activity, respectively. These indices have been repeatedly shown to reflect 5‐HT and DA release and/or metabolic activity.

### 2.13 Aromatase Activity Assay

A tritiated water assay was used to measure aromatase activity (*n* = 7 for each group), with a blind procedure, from whole brains and gonads as previously performed with 1β [3H] androstenedione (CAS number: 63‐05‐8) as substrate (Charlier et al. 2011). Briefly, tissues were homogenized on water‐ice in TEK buffer (150 mM KCl, 10 mM Tris base, and 1 mM EDTA pH 7.2). 50 μL of the homogenate was added to 50 μL of NADPH (1.2 mM final concentration) and 50 μL of 1β [3H]‐androstenedione (25 nM final concentration). We also performed negative controls for each sample with 5 μL of ATD (10^−6^ M final, CAS number: 633‐35‐2), an aromatase inhibitor. The reaction was performed at 37°C for 15 min and stopped by 400 μL of trichloroacetic acid‐charcoal. Samples were centrifuged at 3500 *g* for 15 min at room temperature. Supernatants were transferred to columns containing resin AA. After 3 washes with water, the fractions were collected and mixed with scintillation fluid (4 times the volume of the sample) for radioactivity counting for 1 min. The counts per minute obtained with ATD were deducted from the cpm of the duplicates.

### 2.14 Bulk RNA Barcoding Sequencing (BRB‐Seq)

#### 2.14.1 RNA Extraction

RNA extraction was performed in a blinded procedure on three different parts of the brain, corresponding to the OB, the Tel, and the hypothalamus. Three zebrafish of the same genotype and sex were pooled for each region. A total of 6 pools of 3 wild‐type males and females were obtained (*n* = 18 females and 18 males), 5 pools of 3 mutant males (*n* = 15 males), and 4 pools of 3 mutant females (*n* = 12 females). Samples were homogenized with a mixer pellet pestle (UGAP, cat. no. 2608725), and total RNA was extracted according to the manufacturer's protocol (Macherey‐Nagel, cat. no. 740406.50). RNA quantity and quality were assessed using a Nanodrop‐1000 spectrophotometer (NanoDrop technology, Cambridge, UK) and a 2100 Bioanalyzer Instrument (Agilent Technologies, CA, USA) following the manufacturer's instructions. Only samples with an RNA integrity number (RIN) score > 7 were included.

#### 2.14.2 Library Preparation and Sequencing

Transcriptome analysis was performed in a blinded procedure using 3′ BRBseq with library preparation as described by Alpern et al. (2019). Briefly, reverse transcription and template switching were carried out on 4 μL of total RNA at a concentration of 2.5 ng/mL. The resulting cDNA was purified and amplified to double‐stranded cDNA through PCR. We used 50 ng of the ds cDNA for library preparation via tagmentation using the Illumina Nextera XT kit (Illumina, cat. no. FC‐131‐1024), following the manufacturer's protocol. Sequencing was then performed on an Illumina NovaSeq system by the IntegraGen company, following standard Illumina procedures. Image analysis and base calling were done using RTA 2.7.7 and bcl2fastq 2.17.1.14, with adapter dimer reads removed using DimerRemover (https://sourceforge.net/projects/dimerremover/).

#### 2.14.3 Data Preprocessing

The preprocessing of raw data is described in detail in Giacosa et al. (2021). The quality control pipeline involved an initial read of 16 bases with a quality score > 10, where the first 6 bp were unique to the sample and the subsequent 10 bp represented a unique molecular identifier (UMI). The second reads were aligned to a zebrafish reference transcriptome (UCSC, release danRer11, downloaded in August 2020). Reads mapping to multiple genomic locations were excluded from further analysis. A gene count matrix was then generated by counting the number of unique UMIs associated with each gene (rows) across samples (columns). This matrix was subsequently normalized using the rlog transformation from the DESeq package (Love et al. 2014). All raw and preprocessed data are available in the GEO repository (GEO accession number: GSE299412).

#### 2.14.4 Differential Gene Expression Analysis

Statistical filtering was performed using R v4.0.3. Principal component analysis (PCA) was conducted using the FactoMineR package in R (Lê et al. 2008). Differentially expressed genes (DEGs) were identified by comparing control and mutant groups for each sex and tissue (olfactory bulbs, telencephalon, and hypothalamus). Additionally, we sought to identify genes with sexually differential expression by comparing male and female samples within each model (control or mutant) and for each tissue. Two rounds of filtering were applied to each comparison: first, genes with fewer than three raw counts were excluded, followed by the exclusion of genes not expressed above a defined background threshold for the normalized data, corresponding to the median value (1.049 for the three‐brain‐region dataset) of the entire normalized expression matrix. Second, genes with less than a 1.5‐fold change compared to controls were filtered out. Final statistical filtering was conducted using the LIMMA package (Smyth 2004). Statistically significant DEGs were identified using a cut‐off value of *p* = 0.05, adjusted for multiple testing with the Benjamini & Hochberg method (Benjamini and Hochberg 1995).

#### 2.14.5 Clustering Analysis

Partitioning of the DEGs was performed by using the k‐means method. The resulting expression patterns of DEGs were displayed as a false‐color heatmap using the pheatmap package (Kolde et al. 2018; https://github.com/raivokolde/pheatmap). Each tile is colored with a different intensity according to the log2 fold change. In addition, the dataset is rearranged in each dimension of the mosaic according to the dendrograms calculated for samples and genes, respectively; 13 patterns have been defined to group genes that behave in the same way according to the dendrograms.

#### 2.14.6 Functional Analysis

Functional analysis was conducted using the Annotation Mapping Expression and Network (AMEN) suite (Chalmel and Primig 2008). Annotation terms were considered enriched when the False Discovery Rate (FDR)‐adjusted *p*‐value was ≤ 0.05 and when the number of DEGs associated with each term exceeded five.

### 2.15 GO Biological Process Annotation of DEGs

In males and females with *cyp19a1b*‐mutant vs. WT, for each brain area (OB, Tel, and hypothalamus), we used the Cytoscape software (v3.10.1) with the StringApp plugin (v2.2.0) to create networks of DEGs with a functional score of 0.7. For all six networks created, one by brain area for males and females, we grouped some DEGs according to selected biological processes terms from the Gene Ontology, such as population cell proliferation, neurogenesis, axonogenesis, gliogenesis, synaptic signaling, social and locomotory behavior, steroid metabolic process, and signaling.

### 2.16 Statistical Analyses

#### 2.16.1 Embryo Data (Body Length, Eye Size, Swim Bladder Size, and Heart Rate), Behavioral Data, and Reproduction Data

Raw data were exported as Excel files, and analyses were performed using R (v 4.3.2) via RStudio (v 2023.09.1 + 494). For behavioral data, video recording was checked, and poor quality trackings were either edited manually or discarded. Normality of data distribution was evaluated via quantile‐quantile graphics. Possible outliers were removed based on the interquartile criterion (IQR): values lying above or below 1.5*IQR were discarded (less than 10% of the data). *T*‐tests were applied to embryo data (heart rate, swim bladder size, eye size), shoaling data, and reproduction data (reproductive behavior and spawn characterization), with the exception of spawn success, which was evaluated using a Chi‐2 test. For the rest of the behavioral data, sex was taken into account as another factor, and two‐way (sex, genotype) ANOVA followed by Tukey's post hoc test was performed to identify differences between groups. In parallel, PCA was performed on the entire behavioral dataset (50 observations vs. 20 variables; package factoextra). Only significant PC axes and variables showing significant contribution on these axes were considered for interpretation of the data (except when mentioned otherwise; Camargo 2022). ANOVA followed by Tukey's post hoc test was performed on significant PC scores. An adjusted *p*‐value threshold of 0.05 was set to report statistically significant differences between groups.

#### 2.16.2 Embryonic Survival, Sex‐Ratio Data, Immunostaining Analyses, Monoamines and Metabolites, and Aromatase Activity Assay

Statistical analyses were performed in GraphPad Prism (v. 9.5.1) and in RStudio (v. 1.4.1106.0) for the Sheirer‐Ray‐Hare test. The effect of genotype and day post‐fertilization on survival was tested with two‐way repeated‐measures ANOVA. The effect of genotype on sex ratio was tested using a Student's *t*‐test. For immunostaining and monoamines and metabolites analyses, two‐way (sex and genotype) ANOVA was used for each brain area, each immune‐labeling, and each neurotransmitter and its metabolites. For the aromatase activity assay, the counts per minute (cpm) obtained with the ATD were deducted from the cpm of the duplicates. Two‐way (sex and genotype) ANOVA was used for brain and gonad. All data were tested for normality with the Shapiro–Wilk test and equal variance with Levene's test. If the data did not follow a normal distribution, data were transformed (logarithm of 2). If it was not sufficient, a Sheirer‐Ray‐Hare test was performed. An adjusted *p*‐value ≤ 0.05 was considered a statistically significant difference and was indicated by stars on figures. Hashtags indicate a trend between genotypes (0.05 < *p*‐value ≤ 0.1).

## 3 Results

### 3.1 Characterization of the *cyp19a1b*‐Mutant Line

**FIGURE 1:**
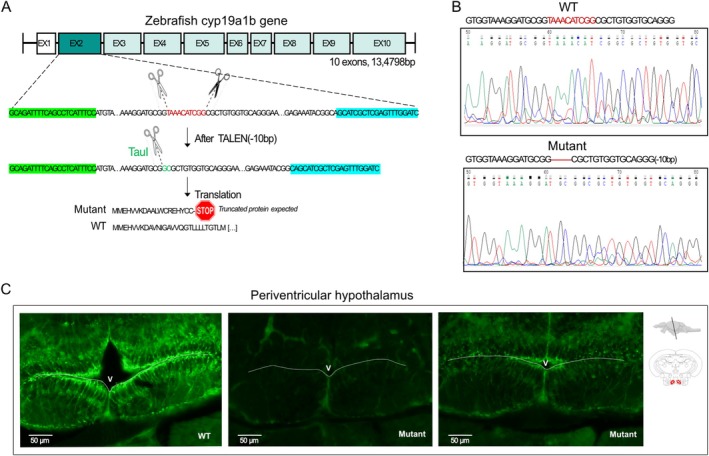
C*yp19a1b* mutant zebrafish characterization. (A) The position of the 10 deleted nucleotides is shown on the top (red sequence), and the corresponding truncated protein is indicated below the gene sequence. The restriction enzyme cut site used for genotyping is written in green (*TauI*). Primer sequences used for genotyping are highlighted in green (forward) and blue (reverse). (B) Sequencing of the *cyp19a1b* gene in WT and mutant zebrafish showing the nucleotide deletion in exon 2. (C) Periventricular hypothalamus sections of AroB immunoreactivity in WT and mutant fish. Scale bars: 50 μm.

To understand the role of brain aromatase in neuroplasticity and behavior, we generated a zebrafish mutant line in which the *cyp19a1b* gene was disrupted using TALENs technology. The deletion created a new recognition sequence for the *TauI* restriction enzyme, which was then used for genotyping (Figure 1A). Sequencing of the *cyp19a1b* gene confirmed the 10‐nucleotide deletion in exon 2, inducing an open reading frame shift and the occurrence of a premature stop codon in the sequence (Figure 1B). Strong AroB immunofluorescent staining was observed in radial glial cells bordering the ventricles in WT zebrafish brains, as shown in the caudal hypothalamus (Figure 1C). In mutant fish, different labeling intensities were observed, albeit consistently reduced compared to the WT. Mutant fish either showed a complete absence of AroB staining compared to WT or a weak fluorescence intensity (Figure 1C).

**FIGURE 2:**
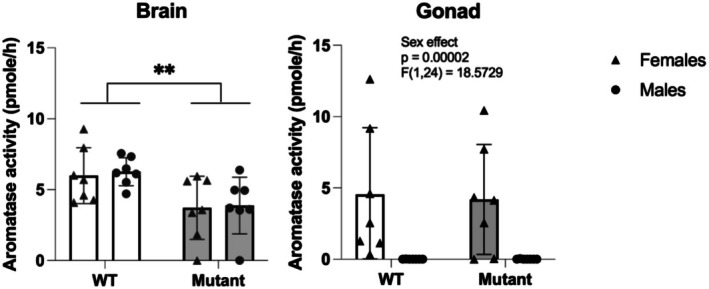
Aromatase activity expressed in pmole per hour in the brain and the gonad of WT and mutant adult zebrafish, in males and females. *n* = 7 in each group. Mean ± SEM; **p < 0.001 versus WT group.

To further characterize the impact of the mutation, we measured aromatase activity and showed significant reductions in the brains of both female and male mutants compared to WT, independently of the sex (*F* (1,24) = 11.01; *p* < 0.01; Figure 2). As predicted, the mutation did not affect aromatase activity in the gonads in the mutant compared to WT, but there was a strong sex effect in the gonads, with strong aromatase activity in the ovaries while no enzymatic activity was detected in the testicles (*F* (1,24) = 18.5729; *p* < 0.001; Figure 2). Together, these results indicate that the nucleotide deletion into exon 2 of the *cyp19a1b* gene induces a significant decrease in aromatase enzyme activity in the brain of mutant females and males, without impacting gonadal aromatase enzyme activity.

### 3.2 Development and Growth

There was no significant difference in percentage survival between WT and mutant larvae (Figure S1A). Similarly, heart rate frequency at 48 hpf showed no significant variation between mutant and WT zebrafish. Furthermore, at 120 hpf, total body length, swimbladder surface, and eye size were similar between mutant and WT individuals (Figure S1B). AroB mutants and WT fish did not significantly vary in length or weight at adulthood. Males were significantly lighter than females independently of the genotype (*F* (1,46) = 8.82; *p* < 0.01). No difference in sex ratio was observed between WT and mutant fish (Figure S1C).

### 3.3 Non‐Reproductive Behaviors

A complete overview of the results of all the behavioral tests carried out is given in Table S2.

#### 3.3.1 Genotype‐Dependent Effects

**FIGURE 3:**
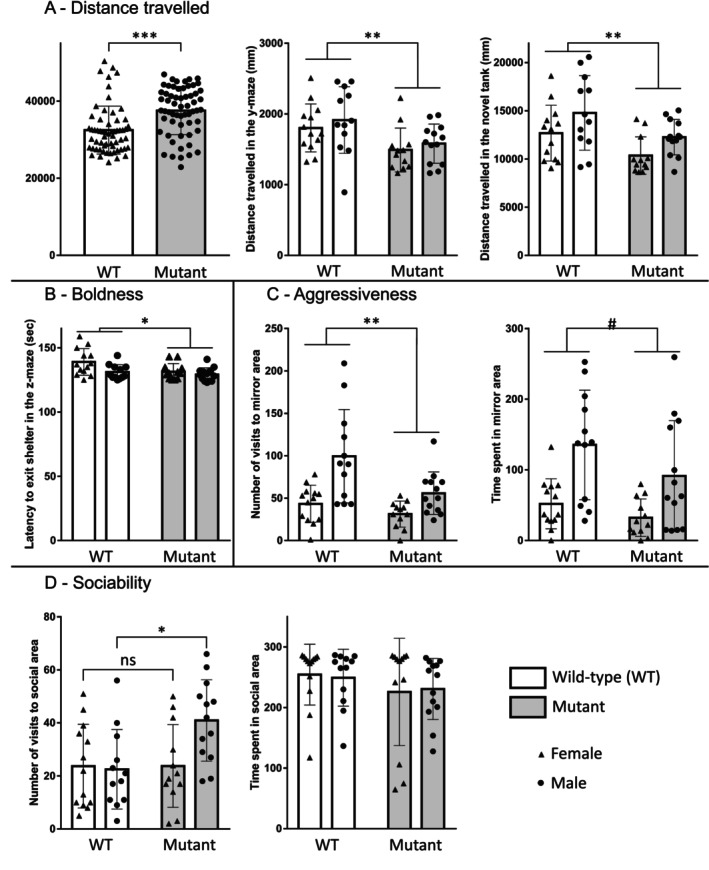
Non‐reproductive behavior in WT and mutant adult fish: Distance traveled in the shoaling test, the y‐maze, and the novel tank diving test (A). Boldness measured by the latency to exit the shelter area in the z‐maze (B). Aggressiveness test with the number of visits and the time spent in the mirror area (C). Sociability is measured by the number of visits and the time spent in the social area (D). Data are shown as mean ± SEM. *p < 0.05; **p < 0.01; ***p < 0.001; #*p* < 0.1 versus the WT group.

Mutant fish exhibited increased activity in the shoaling test (*t*_122_ = 4.46; *p* < 0.001; Figure 3A). However, in behavioral tests assessing individual fish behavior, such as the y‐maze and the novel tank diving test, mutant fish showed reduced swimming activity (*F* (1,46) = 9.98; *p* < 0.01 and *F* (1,46) = 9.10; *p* < 0.01, respectively; Figure 3A). Swimming activity differences between mutant and WT fish are dependent on the context. In the z‐maze test, mutant fish took less time to exit the shelter area, a proxy to evaluate boldness (*F* (1,46) = 6.28; *p* = 0.016; Figure 3B). Mutant fish are less aggressive than WT. They went less often to the mirror area than WT counterparts (*F* (1,46) = 8.02; *p* < 0.01; Figure 3C). There was a genotype (*F* (1,46) = 8.02; *p* < 0.01) and a sex effect (*F* (1,46) = 18.97; *p* < 0.001). In addition, mutant fish tended to spend less time overall in the mirror area compared to WT fish (*F* (1,46) = 2.98; *p* = 0.091; Figure 3C). Mutant fish tended to have a lower spatial memory and to be less anxious than WT, as they tended to spend less time in the novel arm during the y‐maze test (*F* (1,46) = 2.84; *p* = 0.099; Figure S3A) and to spend more time in the top zone in the novel tank diving test (*F* (1,46) = 3.30; *p* = 0.076; Figure S3B).

In summary, mutant animals exhibit increased activity in group settings but reduced activity when alone. They display greater boldness and lower levels of aggressiveness. Finally, they tend to show reduced spatial memory and decreased anxiety‐like behavior.

#### 3.3.2 Sex‐Dependent Effects

Sex‐specific analysis across the different tests reveals that males covered a greater distance than females in most of them: Z‐maze (*F* (1,46) = 22.42; *p* < 0.001), novel tank diving test (*F* (1,46) = 6.89; *p* = 0.012), sociability tests (*F* (1,46) = 5.34; *p* = 0.025), and aggressiveness tests (*F* (1,46) = 18.50; *p* < 0.001; Table S2). In the z‐maze, males take significantly less time to reach the different zones than females: latency to reach zone 16 (*F* (1,46) = 13.55; *p* < 0.001), latency to reach distal zone (*F* (1,46) = 6.04; *p* = 0.018), time spent in distal zone (*F* (1,46) = 5.63; *p* = 0.022). In the novel tank diving test, the top zone is visited more by males (*F* (1,46) = 6.83; *p* = 0.012). In the aggressiveness test, the number of visits to the mirror zone was higher for males (*F* (1,46) = 18.97; *p* < 0.001), and they spent more time in the mirror zone (s) (*F* (1,46) = 17.93; *p* < 0.001). In short, some behaviors differ according to the sex of the fish, with males swimming more, being bolder and more aggressive, and being less anxious than females.

#### 3.3.3 Genotype and Sex Interaction

Two significant genotype–sex interaction effects were observed. Mutant males appeared to be more sociable, as they visited the social area more often than both WT fish and mutant females (*F* (1,46) = 4.42; *p* = 0.041; Figure 3D). There was no difference for WT fish, whatever the sex, and no difference for mutant females compared to WT fish (Figure 3D). In the classical conditioning test, there is an interaction between genotype and sex for the number of visits to the checkerboard (*F* (1,46) = 6.19; *p* = 0.017; Table S2), but differences are small, and the post hoc does not reveal significant differences.

### 3.4 Reproductive Behavior

We quantified various locomotor parameters to evaluate sexual behavior and found no difference in distance traveled during reproduction, time spent in the spawning area, and average interindividual distance. Body contact time tended to be reduced for mutant fish compared to WT fish (*t*_81_ = −1.67; *p* = 0.098; Figure S2A). Nonetheless, the number of eggs laid per individual spawn, the success frequency (data not shown), and the fertilization rate were similar between both genotypes (Figure S2B).

### 3.5 Brain Proliferation

**FIGURE 4:**
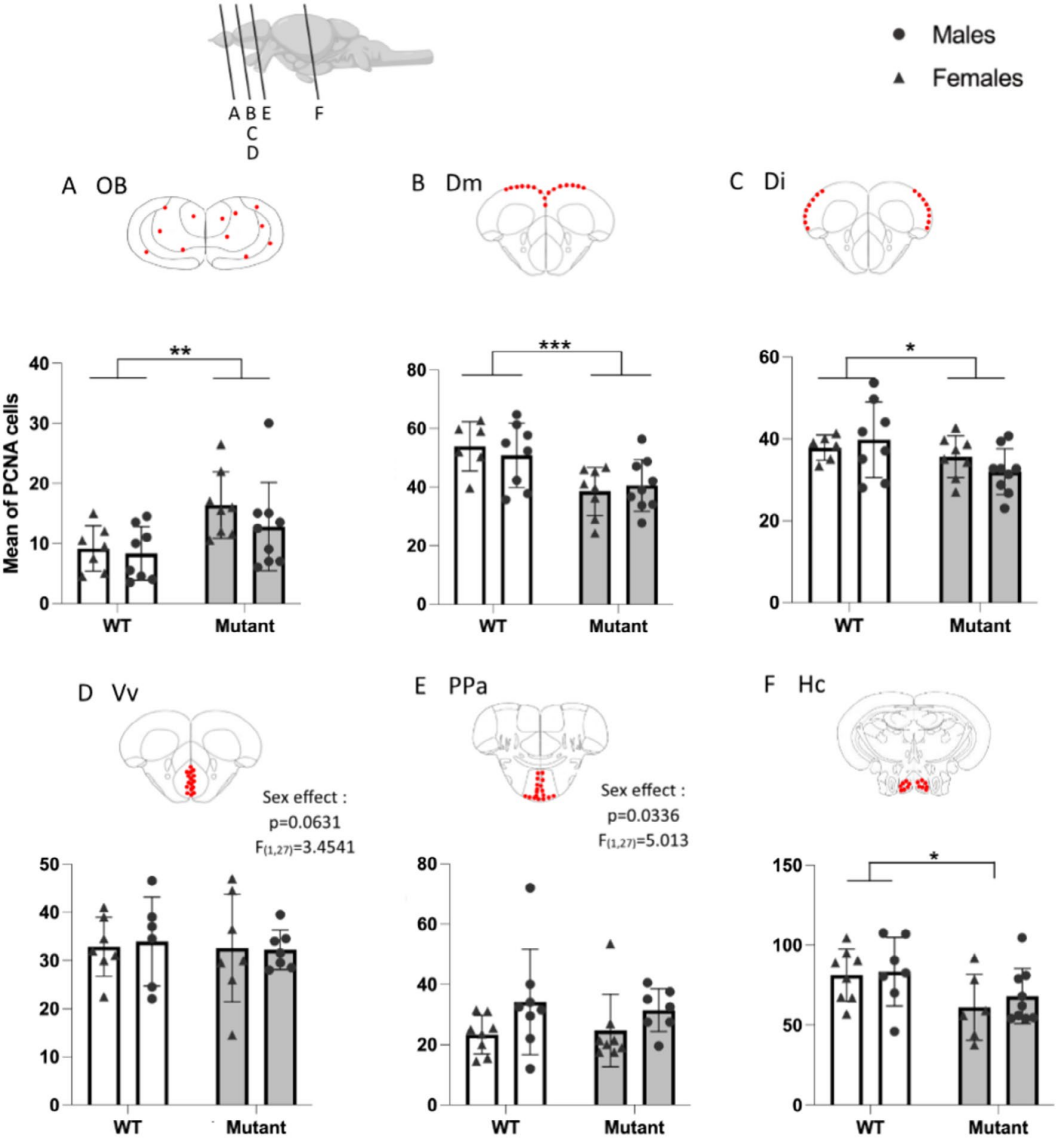
Quantification of the number of PCNA‐labeled cells in WT and mutant fish. (A) Olfactory bulbs (OB). (B) Medial zone of dorsal telencephalic area (Dm). (C) Lateral zone of dorsal telencephalic area (Di). (D) Ventral nucleus of ventral telencephalic area (Vv). (E) Parvocellular preoptic nucleus (PPa). (F) Caudal zone of periventricular hypothalamus (Hc). Red dots on schematic frontal sections indicate quantified areas. Mean ± SEM, *p < 0.05; **p < 0.001; ***p < 0.0001 versus WT group.

Cell proliferation was evaluated by counting the number of PCNA‐labeled cells. In the OB, cell proliferation was significantly higher in mutant fish compared to WT, independently of sex (*F* (1,28) = 9.95; *p* < 0.01; Figure 4A). In contrast, proliferation was significantly reduced in mutants compared to WT in the Dm (*F* (1,27) = 14.58; *p* < 0.001; Figure 4B), the Di (*F* (1,27) = 4.738; *p* = 0.0384; Figure 4C), and the Hc (*F* (1,26) = 6.528; *p* = 0.0168; Figure 4F). Additionally, a higher number of PCNA‐labeled cells was observed in males compared to females in the PPa, independently of genotype (*F* (1,27) = 5.013; *p* = 0.0336; Figure 4E). A similar trend was observed in the Vv (*F* (1,27) = 3.4541; *p* = 0.0631; Figure 4D). No statistically significant difference was found in other regions investigated (D, thalamus, Hv, Hd, and pituitary; Figure S4).

### 3.6 Dopamine and Serotonin‐Producing Neurons

**FIGURE 5:**
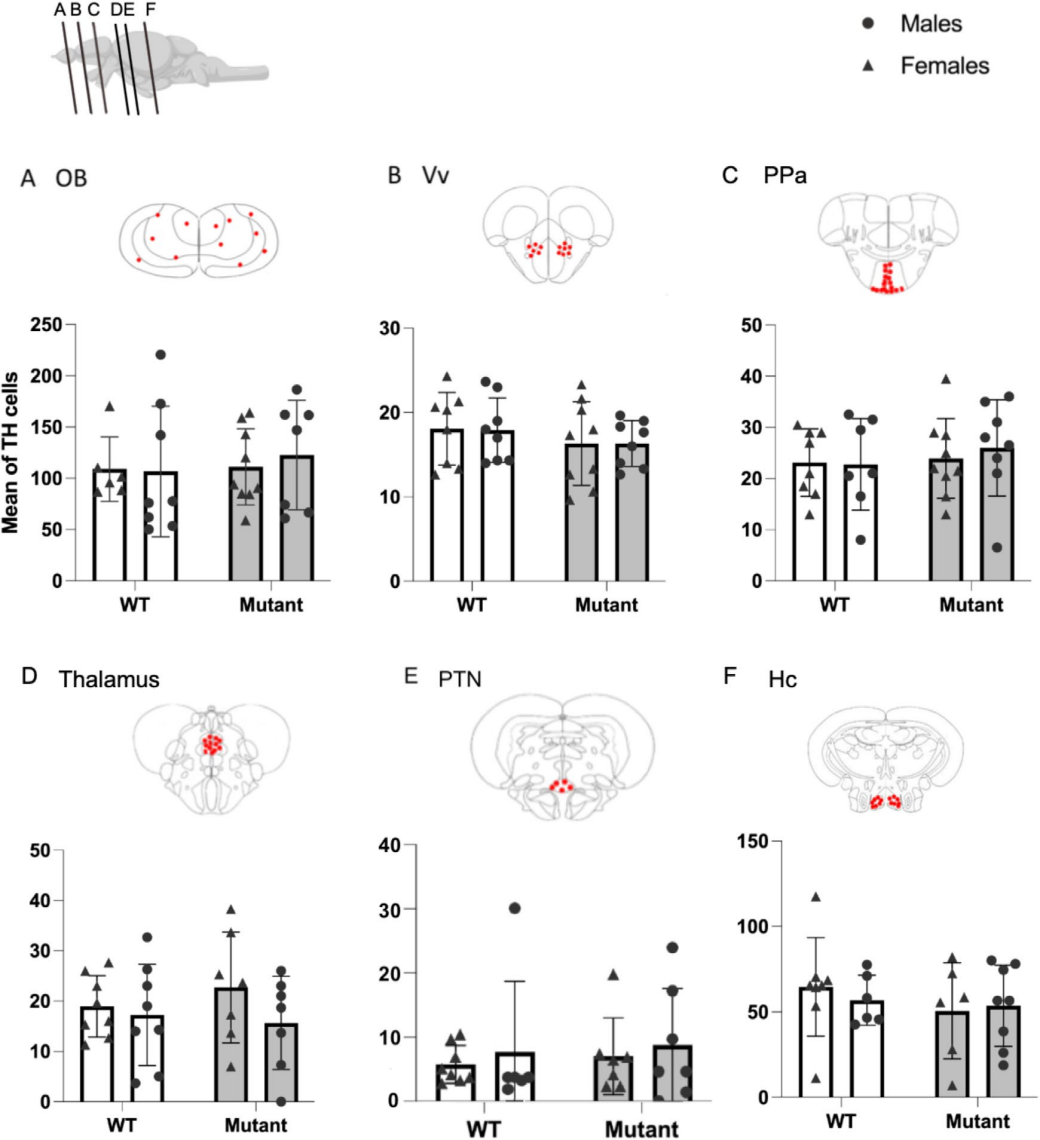
Quantification of TH‐positive cells in WT and mutant fish. (A) Olfactory bulbs (OB). (B) Ventral nucleus of ventral telencephalic area (Vv). (C) Parvocellular preoptic nucleus (PPa). (D) Thalamus. (E) Posterior tuberal nucleus (PTN). (F) Caudal zone of periventricular hypothalamus (Hc). Red plots represent regions of interest in which TH‐positive cells have been quantified. Mean ± SEM; the number of analyzed brains is indicated in each bar.

In all neuronal populations examined, no significant difference in the number of TH‐labeled cells was observed between WT and mutant zebrafish (Figure 5).

**FIGURE 6:**
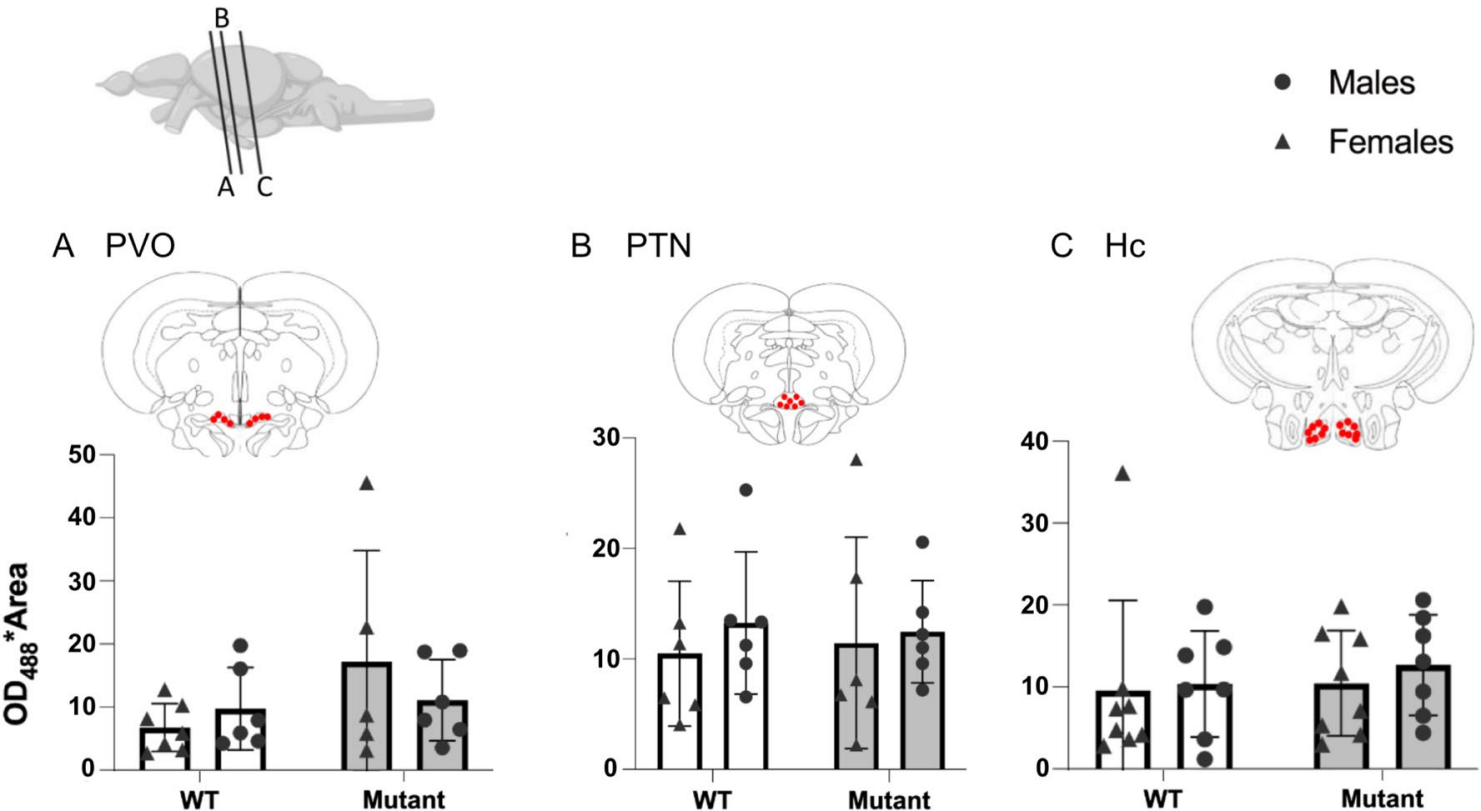
Expression of 5‐HT in positive cells in WT and mutant fish. (A) Paraventricular organ (PVO). (B) Posterior tuberal nucleus (PTN). (C) Caudal zone of periventricular hypothalamus (Hc). Red dots on frontal brain section drawings indicate areas of interest where expression is observed. Mean ± SEM.

The optical density of 5‐HT positive cells was quantified in the paraventricular organ, the posterior tuberal nucleus, and the caudal zone of the periventricular hypothalamus. No significant difference in 5‐HT expression was observed between WT and mutant zebrafish (Figure 6).

In parallel to the histological quantification of cell‐producing neurotransmitters, we also measured the levels of dopamine, serotonin, and noradrenaline and their metabolites in the whole brains of WT and mutant male and female fish. A complete overview of the results can be found in Table S3. A modulation of 3MT was observed in mutant fish, with mutant females having significantly more 3MT than WT females (*t* = 0.0464, Table S3).

### 3.7 Transcriptomic Analysis

**FIGURE 7:**
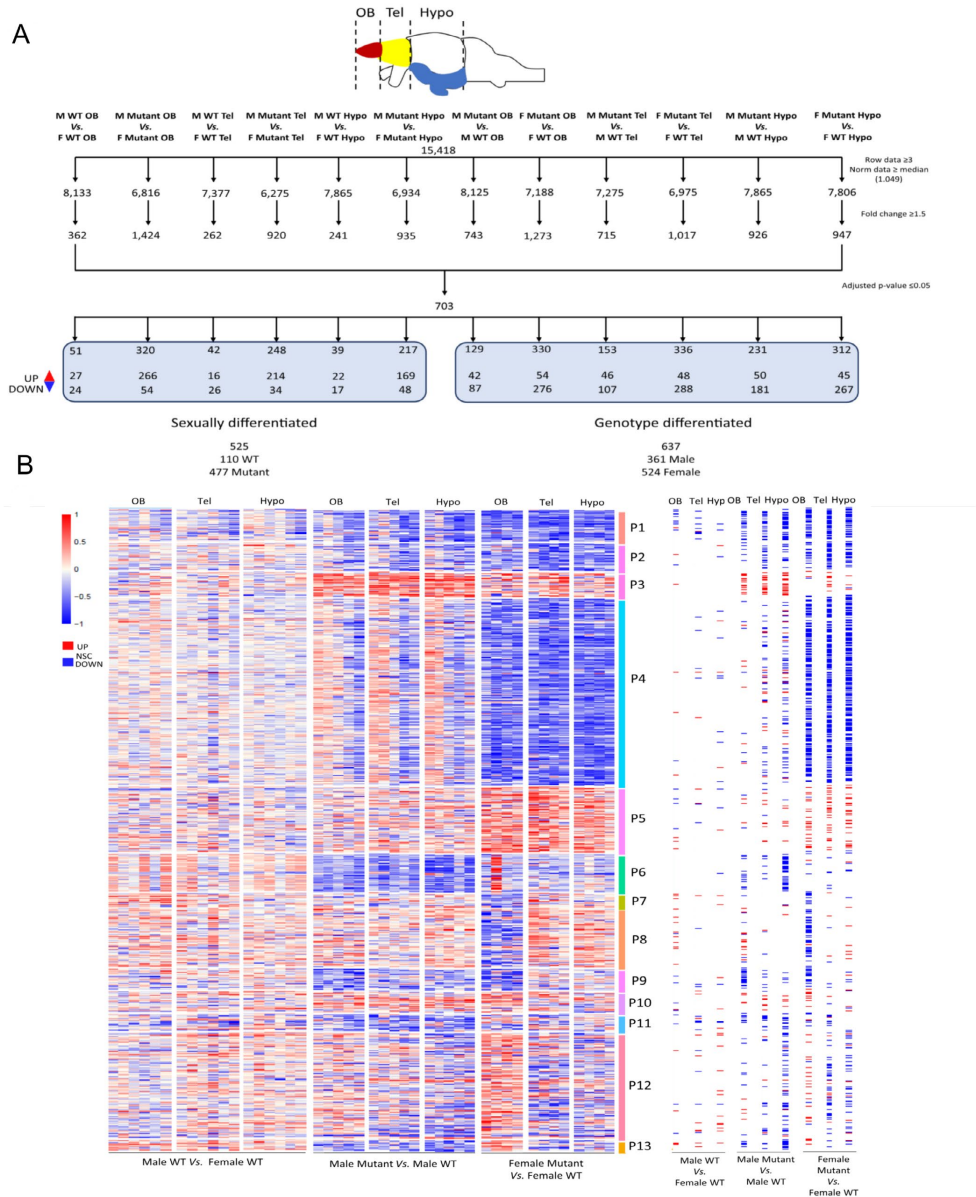
Workflow for BRB‐Seq data processing and heatmap of differentially expressed genes (DEGs) in the three different parts of the brain (olfactory bulb, telencephalon, and hypothalamus) on male and female WT and mutant adult fish. (A) Filtration steps based on limit of detection, fold change cut‐off of 1.5, and statistics leading to 703 DEGs. Hypo, hypothalamus; OB, olfactory bulb; Tel, telencephalon. (B) Heatmap of all DEGs in each WT and mutant fish in the three areas of the brain. Genes are clustered in 13 distinct patterns: P1. 36 DEGs, P2. 31 DEGs, P3. 27 DEGs, P4. 213 DEGs, P5. 74 DEGs, P6. 43 DEGs, P7. 15 DEGs, P8. 66 DEGs, P9. 24 DEGs, P10. 23 DEGs, P11. 19 DEGs, P12. 120 DEGs, P13. 12 DEGs. On the right‐hand side of the heatmap, each dysregulated gene is represented as a tile, with a color intensity reflecting the average log2 fold change of samples for each comparison and brain region. Scale: Log2 fold change. Red = significantly upregulated expression, Blue = significantly downregulated expression, White = no significant change. Male: *N* = 6 WT and 5 mutants; Female: *N* = 6 WT and 4 mutants.

Differential transcriptomics analyses were carried out to compare DEGs in males vs. females and mutants vs. WT in the OB, the Tel, and the hypothalamus. A filtering pipeline was applied to the results to select significant DEGs only (Table S4, Figure 7A). DEGs can be analyzed from transcriptome data by visualizing expression profiles using a heatmap. This approach allows quick identification of gene groups exhibiting up‐ or downregulation across different genotypes, sexes, or brain regions. Analysis of the clustered expression profiles identified 13 distinct patterns labeled P1 to P13 (Figure 7B). A GO term enrichment analysis was performed for each pattern but failed to produce significant results.

We identified 110 DEGs between WT males and females (Figure 7A). However, the clustering did not link these DEGs to any of the specific patterns identified (Figure 7B). In contrast, sex‐related differences were more pronounced in mutants, with 477 DEGs identified (Figure 7A).

A total of 637 genotype‐dependent DEGs were identified, with female and male mutants exhibiting 524 DEGs and 361 DEGs, respectively, when compared to wild‐type counterparts (Figure 7A). Across the three regions analyzed, genotype‐dependent DEGs were generally downregulated in mutants relative to WT, an effect more pronounced in females than in males (Figure 7B). When mutants were compared to WT, we observed that genes expressed in patterns P1, P2, P4, and P5 were differently affected by the mutation according to sex. Specifically, genes in P1, P2, and P4 were downregulated in both sexes, but the effect was more pronounced in mutant females than in mutant males. In contrast, gene expression in P5 was increased in mutant females compared to mutant males (Figure 7B).

For each region and for each sex, we identified genes affected by the mutation. We annotated them according to Gene Ontology, under the category “biological processes,” and looked for genes containing annotations of interest. We then submitted this dataset to a STRING analysis to build interactions between partner genes and visualized these interaction networks and biological pathways using the Cytoscape software, integrating these networks with annotations and gene expression profiles (Figures S5, 8, and 9).

**FIGURE 8:**
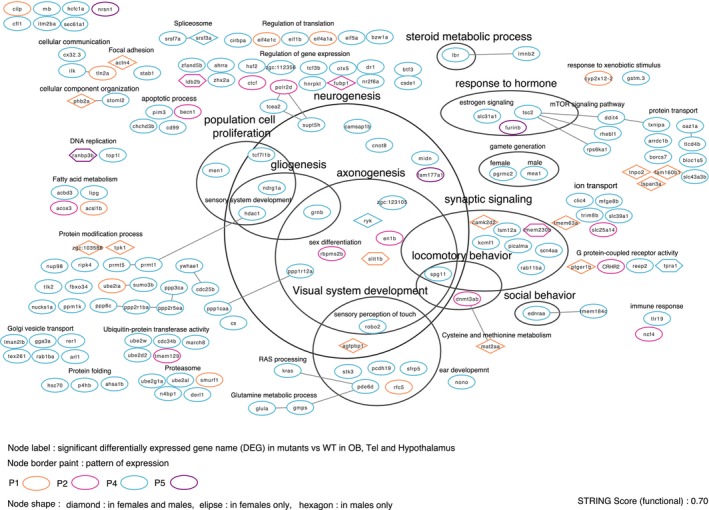
Functional interaction and annotation of genes from patterns (Figure 7B) P1, P2, P4, and P5 (indicated by the node border color) are significantly differentially expressed in mutants versus WT in the three brain areas, in males and/or in females (indicated by the node shape).

**FIGURE 9:**
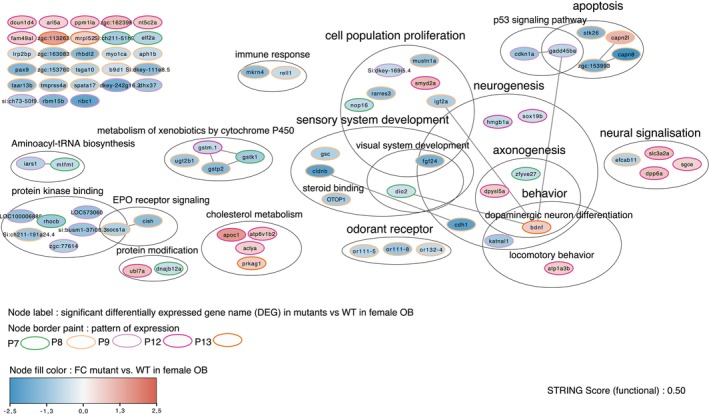
Cytoscape network visualization: Functional interaction (STRING functional score: 0.50) and annotation of genes from pattern of expression (described in Figure 7B and indicated by the node border color) P7, P8, P9, P12, and P13 (indicated by the node border color), significantly differentially expressed in female mutant versus WT, in the olfactory bulbs (OB).

Figure S5 shows the 6 resulting networks, for males and females, mutants vs. WT, in each of the three brain areas. This representation provides a quick overview of the mutation's impact on gene expression, as indicated by color intensity based on log2 fold change and highlights the different biological processes associated with the dysregulated genes. The networks in Figure S5 display the names of DEGs, their dysregulation levels, and their localization. The color code and the node shape provide further information: Diamond‐shaped nodes indicate genes common to both sexes and all brain regions, while green borders represent sex‐specific genes shared by all three brain areas.

We focused on DEGs known to be involved in the biological processes of interest, such as cell population proliferation (*men1, rarres3, hdac1, kctd13*), neurogenesis (*spg11, hdac1, bdnf, robo2, agtpbp1, supt5h, hmgb1a, kctd13, sema5a*), axogenesis (*spg11, bdnf, robo2, sema5a, pak1*), gliogenesis (*hdac1*), neurotransmission (*gabra4, npas4a, rab3a*), steroid metabolism (lbr, *dhcr7*), sensory systems development (*pcdh5, sfrp5, pde6d, rfc5, nono, or111, or132*), cognition (*robo2, bdnf*), and behaviors (*bdnf, psmd3, dnmt3ab, spg11, ednraa, npas4a*). Beyond these processes, we also highlighted a significant dysregulation of genes involved in transcription, protein translation, folding, and degradation.

In this study, we also closely examined the expression patterns on the heatmap that differed according to genotype and sex on the heatmap (Figure 7B). DEGs contained in patterns P1, P2, P4, and P5 that were significantly impacted by the mutation in males and/or females (mutant vs. WT) across all brain regions are presented in Figure 8. Fifteen of these genes were shared by males and females (diamond node shape, Figure 8), with the most dysregulated including *agtpbp1* (neurogenesis/visual development), *actn4* (focal adhesion), *tnpo2, tspan3a* (protein transport), and *camk2d2* (synaptic signaling). Most of the dysregulated genes were female‐specific and exhibited a greater response to the mutation compared to males (Figure S5, ellipse node shape, Figure 8). Among them, a large proportion was downregulated, notably *kcmf1* (synaptic signaling), *nono, stk3, sfrp5, pde6d* (sensory development), *spg11, robo2* (sensory perception/locomotory behavior), *men1*, and *tcf7l1b* (cell population proliferation), and *supt5h, camsap1b*, and *cnot8* (neurogenesis), and *grnb, rbpms2b, en1b*, and *ppp1r12a* (axonogenesis). Five DEGs were specific to males (hexagon node shape, Figure 8) but were only moderately dysregulated (*ranbp3b*, *tpra1, ldb2b, slit1b, tmem232*).

We also observed that, in mutant females compared to WT females, gene expression in OB was differentially impacted by the mutation compared to the telencephalon and hypothalamus. These DEGs, corresponding to the patterns P7, P8, P9, P12, and P13, were shown in the functional network depicted in Figure 9. Genes associated with patterns P7, P8, and P9 were notably downregulated in OB and should play a role in immune response, metabolism of xenobiotic cytochrome P450, cell population proliferation, apoptosis, neurogenesis, protein kinase binding, sensory system development, and odorant receptors.On the other hand, genes associated with patterns P12 and P13 were upregulated in mutant females OB and could be involved in neural signalization, behavior (locomotory and cognition), and cholesterol metabolism.

Proteins investigated by immunofluorescence, such as PCNA, were identified by transcriptomics, as were enzymes involved in the metabolism of monoamines (COMT, MAO, and TH). *PCNA* transcript was downregulated in the olfactory bulbs of mutant males (Figure S5). No differences in gene expression were observed between mutant and wild‐type fish for other proteins.

## 4 Discussion

This study aimed to characterize the role of brain aromatase in regulating reproductive and social behavior as well as its influence on brain proliferation and dopaminergic and serotonergic neurotransmission. Our results revealed a sex difference across multiple behavior traits, notably locomotor activity, social interaction, aggressiveness, and anxiety. Moreover, the *cyp19a1b* gene mutation was found to exert a context‐dependent influence on locomotor activity, enhance boldness, and reduce aggressive behaviors. Additionally, the mutation impacted cell proliferation in a region‐specific manner. Transcriptomic analysis identified widespread gene expression alteration in both male and female mutant zebrafish, with a greater effect in females. They also revealed a mutation‐specific impact on gene expression in olfactory bulbs in females, which was not observed in males.

### 4.1 Cyp19a1b Mutant Line Characterization

The successful mutation of the *cyp19a1b* gene was confirmed by sequencing and genotyping. However, the mutation did not lead to a complete loss of brain aromatase, as weak Aromatase B immunostaining and enzymatic activity were still detected in mutant zebrafish. A first hypothesis would be that the decrease of brain aromatase protein, induced by the mutation, triggers a genetic compensatory process leading to an upregulation of the *cyp19a1a* gene in the brain, but our transcriptomic data did not support this hypothesis. In addition to genetic compensation, other surveying mechanisms may help mitigate the loss of function induced by the mutation. In zebrafish, cryptic transcription—the production of non‐canonical transcripts—has been shown to promote the expression of alternative transcripts. Depending on the position of the cryptic site used, functional transcripts may still be generated and lead to the production of alternative functional protein isoforms (Anderson et al. 2017; Rouf et al. 2022). In our mutant model, a cryptic transcription could have been activated, leading to the synthesis of a truncated aromatase isoform that retains its catalytic domain but has reduced enzymatic activity and presents the last 15 amino acid residues recognized by the antibody (Graham‐Lorence et al. 1995).

### 4.2 Brain Aromatase and Non‐Reproductive Behavior

We also examined the implications of brain aromatase on non‐reproductive behaviors. In the present study, behavioral analyses showed that the effect of the *cyp19a1b* mutation on fish activity differed when fish were in a group (shoaling) or alone. Mutant fish were hyperactive in the shoaling test, whereas they exhibited reduced swimming activity in individual setups, such as the novel tank diving test and the Y‐maze test, compared to WT fish. These context‐dependent effects on locomotor behavior may result from the impact of the AroB mutation on other behavioral aspects, such as sociality, boldness and aggressiveness‐traits influenced by the presence of conspecifics. An altered perception of conspecifics could be attributed to a disruption in sensory system development or function, as suggested by our transcriptomic findings revealing dysregulation of several related genes (*agtpbp1, nono, stk3, sfrp5*, and *pde6d*). Previous studies on endocrine disruption have reported hyperactive phenotypes in zebrafish and other models with altered AroB expression (Blanc‐Legendre et al. 2025; Fenske et al. 2020). Some of the behaviors investigated were affected by the AroB mutation in a sex‐specific manner. We observed a significant increase in sociability in male mutant fish. Although a reduction in aggressiveness was noted in both sexes, it was considerably more pronounced in mutant male fish. The role of brain aromatase in modulating aggressiveness is well‐documented across vertebrates, including teleosts (Huffman et al. 2013; Jalabert et al. 2015; Shaw 2018; Trainor et al. 2006). For instance, in various fish species, inhibiting aromatase activity through fadrozole exposure lowered aggressiveness (Huffman et al. 2013; Zubizarreta et al. 2020). Similarly, in zebrafish, exposure to the endocrine disruptor ethinylestradiol led to increased aggressive behaviors (Fenske et al. 2020; Filby et al. 2012), though other findings suggest that androgens may have a stronger modulatory effect on aggressiveness (Liu et al. 2020). Our results on sexually differentiated aggressive behavior and the stronger impact of AroB reduction in males compared to females clearly suggest the importance of local estrogen production in the regulation of aggressiveness. Filby and colleagues observed elevated transcription of genes encoding estrogen receptors (ERβ1, ERβ2) in dominant, more aggressive males, while dominance in females did not appear to be directly linked to estrogenic pathways (Filby et al. 2010). Compared to other species, relatively little is known about the mechanisms underlying steroid‐dependent aggressiveness in zebrafish (Genario et al. 2020). Future research should focus on how sex steroids, from both gonadal and brain origins, modulate aggressiveness toward same‐sex versus opposite‐sex conspecifics. Modulation of aggressiveness should be interpreted in light of the results obtained from the sociality test, as we observed that AroB mutant male fish visited the social area more often than their WT counterparts. However, the total time spent in the social zone was similar in both groups. Interestingly, we recently showed that developmental exposure to EE2 (17α‐ethinylestradiol) led to a significant reduction in several behavioral parameters linked to social interaction, specifically in males, while females were not affected (Blanc‐Legendre et al. 2025). This finding and the present results reinforce the idea that the early organizational effects of brain aromatase/estrogens are crucial for brain programming related to social interactions in zebrafish, as observed in other species (Kellogg and Lundin 1999).

The impacts of the mutation on behaviors we observed in our study are probably the result of simultaneous dysregulations of different sets of genes influencing several behaviors or metabolic pathways, often specific to some brain regions and dependent on the sex. Indeed, some genes involved in cognition and behavior are affected in both males and females. For example, the *gabra4* gene, a GABAergic subunit receptor, is downregulated; interestingly, a previous study suggested that the knockdown of *gabra1* led to hypoactivity (Reyes‐Nava et al. 2020). The dysregulations we highlighted in the transcriptomic analysis do not always occur in the same way in males and females, as exemplified by the *bdnf* and *robo2* genes. The *bdnf* gene, involved in stress response and social memory, is upregulated in females but downregulated in males. Interestingly, a recent study found that *bdnf* knockout zebrafish displayed increased attraction to social stimuli and higher levels of locomotor activity, despite similar swimming capacities (Lucon‐Xiccato et al. 2023). The *robo2* gene, which plays a key role in the development of neural circuits supporting behaviors such as swimming, is upregulated in males and downregulated in females (Tosa et al. 2015). Additional DEGs involved in locomotory behaviors were dysregulated in mutant females only, such as *psmd3* (Fitzgerald et al. 2014), *dnmt3ab* (Lai et al. 2020), and *spg11* (Martin et al. 2012). Another interesting gene, *ednraa*, which encodes the endothelin receptor type Aa known to be involved in behavior and neuroendocrine regulations, is downregulated in mutant females only. It has been shown that zebrafish harboring a mutation in the *ednraa* gene form less cohesive shoals than WT and exhibit increased aggressiveness (Carreño Gutiérrez et al. 2019). On the contrary, the *npas4a* gene, a gene involved in social behavior, fear learning, and adaptive responses to stress, is downregulated but in mutant males only (Baker and Wong 2021; Teles et al. 2016). Overall, the differential transcriptomic analysis comparing mutants and WT clearly demonstrated that the mutation of the *cyp19a1b* gene disrupts the transcription of numerous genes that are likely to influence, either directly or indirectly, the behavioral traits analyzed in this study in a sex‐dependent manner.

Previous work has highlighted the role of AroB in social behavior, possibly through the effects of E2 on sensory perception and communication. Individuals rely on social stimuli to guide their actions and interactions with conspecifics. The expression of aromatase in peripheral sensory organs and sensory afferent fibers is conserved across communication systems (Shaw 2018). Additionally, aromatase and estrogen receptors are present in key sensory integration regions of the brain, where sensory information converges to regulate motor output. In AroB mutant zebrafish, impaired aromatase expression may disrupt the modulation of sensory neuron excitation, altering their firing rates and interfering with input processing across multiple sensory pathways. These disruptions may ultimately contribute to the social behavior changes observed in this study. Our results show that several genes involved in the development and function of sensory systems (visual, olfactory) are affected by the mutation in a sex‐dependent manner (*pcdh5*, *sfrp5*, *pde6d*, *rfc5*, *nono*, *stk3*, *or111‐5*, *or111‐8*, *or132‐4*), probably leading to a differential integration of sensory information between males and females, and ultimately explain the behavioral differences observed between the sexes.

It is interesting to note that some genes linked to the neurodegenerative phenotype are differentially expressed in all mutants, regardless of sex, and in all brain areas. The gene *agtpbp1*, known to coordinate motor activity and associated with the progression of neurodegenerative phenotype in mammals (Baltanás et al. 2021; Kitano et al. 2015), is strongly downregulated in mutant zebrafish, males and females. Notably, several dysregulated genes, involved in neurogenesis, were previously identified as drivers for neurobehavioral phenotypes such as autism spectrum disorder (*kctd13, sema5a*; Ho et al. 2019; Teng et al. 2019) and Parkinson disease (*hdac1*; Pinho et al. 2016; Srivastava et al. 2024) in zebrafish, mammalian models, and/or human. Recently, the development of autism spectrum disorder was suspected to be linked to brain aromatase disruption and has greater incidence in males, do are the related behavioral outcomes observed in the present work (Symeonides et al. 2024).

### 4.3 Brain Aromatase and Reproductive Behavior

Because aromatase in the brain plays a crucial role in the control of reproductive behavior in mammals and birds, especially in males (Aspesi and Cornil 2024; Trainor et al. 2006), we first investigated the impact of reduced AroB protein and enzymatic activity on sex‐related endpoints in zebrafish. In teleosts, the distinct roles of the paralogous genes *cyp19a1a* and *cyp19a1b* remain unclear. *Cyp19a1a* gene expression is fundamental for ovarian development and female sex determination (Yin et al. 2017), and its expression leads to a high aromatase activity in adult ovaries, while no activity is observed in the testis (as shown in the present study). On the other hand, *cyp19a1b* transcription is mostly restricted to the brain and, contrary to *cyp19a1a*, is expressed in both males and females, with no apparent sex difference in brain aromatase activity. We found no differences in sexual behavior or clutch size between mutant and wild‐type zebrafish. While local estrogen synthesis is critical for male sexual behavior in birds and mammals, our findings, along with others, suggest that this may not be the case for zebrafish. Although the exact mechanisms remain to be determined, testosterone or androgen metabolites, rather than estrogens, appear to play a key role in male sexual differentiation and mating behaviors in zebrafish (Dai et al. 2023; Shu et al. 2020). It is important to note that this conclusion may not extend to all teleosts, as certain species, such as guppies and killifish, appear to be affected by central aromatization processes (Roggio et al. 2014; Tian et al. 2015). Additionally, our results regarding female sexual behavior in zebrafish slightly differ from previous studies using a different AroB mutant line. Shaw and colleagues observed delayed initial and last oviposition events and an increased frequency of oviposition in AroB mutant fish, likely due to the mutation's impact on the arginine‐vasopressin signaling pathway (Shaw, Therrien, et al. 2023). According to information provided in the Zfin database for line zf1032 (https://zfin.org/ZDB‐ALT‐180409‐6), the mutation reported in this article produces a frameshift in exon 4, but no information was provided about the consequences of the mutation, especially regarding AroB activity. We, however, acknowledge that in our model, AroB expression is reduced rather than completely absent, which likely contributes to the reduced impact of the mutation.

### 4.4 Brain Aromatase, Neurogenesis, and Neurotransmission

Several studies have demonstrated a link between behavior and neurogenesis. In zebrafish eleutheoembryos, swimming activity, sociability, and brain proliferation were modified after exposure to estrogen‐mimetic molecules (Blanc‐Legendre et al. 2025; Coumailleau et al. 2020; Kinch et al. 2015). We therefore examined proliferation in brain regions that are relevant for the expression of social behaviors (olfactory bulbs, telencephalon, hypothalamus). We highlighted that the decrease of brain aromatase expression induced by the mutation had a region‐dependent impact on cell proliferation in adulthood. Cell proliferation was increased in OB of mutant zebrafish compared to their WT counterparts. However, the medial (Dm) and lateral (Di) zones of the dorsal telencephalic area, corresponding to the amygdala and hippocampus in mammals, respectively, exhibited a significant reduction in cell proliferation. A decrease in proliferation in these key regions, involved in the processing of sensory information, particularly auditory and visual inputs, could be linked to the behavioral alterations observed in mutants, specifically impaired motor activity, sociability, and aggressiveness (Filby et al. 2010; Mueller 2012). Our results also show a significant decrease in proliferation in the caudal hypothalamus, a region known to play a role in aggressiveness. Consequently, impaired proliferation in this area could also be associated with the reduced aggressiveness observed in mutant individuals (Filby et al. 2010).

Previous studies have already shown that estrogens affect cell proliferation and neuronal differentiation in a region‐dependent manner (Diotel et al. 2013, 2018; Makantasi and Dermon 2014). The region‐specific impact of the AroB mutation we highlighted here could be explained by the fact that, although estrogen receptors (both nuclear and membrane‐bound) are expressed in the regions we analyzed (Menuet et al. 2002), their expression levels are variable, potentially leading to differential effects on proliferation. Furthermore, the subcellular co‐expression of different isoforms and different cofactors may also contribute to opposing responses. However, to date, no data are available on the co‐localization of these isoforms in the zebrafish brain. Currently, the identity of cells exhibiting reduced or increased proliferation in mutant fish remains unknown. However, based on their localization in the OB, Dm, Dl, and Hc, they could correspond to radial glial cells/progenitors, neuroblasts, or ependymoglial cells (Byrd and Brunjes 2001; Diotel et al. 2020; Grandel et al. 2006; März et al. 2010). Further investigations will be required to provide more information.

Interestingly, our results indicate that cell proliferation is higher in males than in females in both the ventral telencephalon and the preoptic area, regardless of genotype. While estrogen and androgen receptors are present in these regions (Diotel et al. 2011; Gorelick et al. 2008; Menuet et al. 2002), it remains unclear whether their expression differs between the sexes in zebrafish. In medaka, a marked sex difference was observed in several nuclei of the ventral telencephalic and preoptic areas, where estrogen and androgen receptor expression was prominent in females (Hiraki et al. 2012). In addition, transcriptomics data show sex‐differently modulated genes involved in cell proliferation (*rarres3*, *men1, tcf711b*).

Based on our transcriptomics data, we observed that several genes involved in the maintenance or differentiation of neural stem cells (*supt5h, hmgb1a, camsap1b, cnot8, grnb, ppp1r12a, grnb, en1b*, and *rbpms2b*) were downregulated in mutant zebrafish. AroB mutation also affects the *lbr* and *dhcr7* genes, which are involved in cholesterol synthesis (Miyazaki et al. 2024; Tsai et al. 2016), potentially affecting local steroid production and neurogenesis. The regions are not modulated in the same way by the brain aromatase mutation, and this is accompanied by a difference in gene expression in transcriptomics. The olfactory bulbs have many genes involved in processes such as neurogenesis, cell proliferation, and axonogenesis that are deregulated differently from the telencephalon and hypothalamus.

Our immunofluorescence results do not reveal any impact of the mutation on the number of dopaminergic and serotonergic neurons. In contrast, previous studies in zebrafish larvae have reported effects of E2 or EE2 (an estrogen receptor agonist) on dopaminergic and serotonergic neurons (Nasri et al. 2021; Ulhaq and Kishida 2018). To further complete our results, we quantified the concentrations of dopamine, serotonin, and noradrenaline and their metabolites. Our results do not show major changes in these parameters. However, we observed a significant increase in 3MT levels in the brains of mutant females compared with wild‐type females. 3MT is a dopamine metabolite formed by the enzyme catechol‐O‐methyltransferase (COMT). The increase in 3MT suggests that COMT activity may be higher in the brain of mutant females. At the transcriptomic level, dopamine synthesis, reuptake, metabolism (*COMT* and *MAO*), and receptors were not dysregulated by the mutation. Although our transcriptomic results do not show any changes in dopaminergic and serotonergic transmission in mutant animals, they do highlight an impact on GABAergic neurotransmission, with *gabra4* (GABA receptor alpha 4) downregulated in mutant females’ hypothalamus, and *rab3a*, which is downregulated in mutant females’ telencephalon and in mutant females’ and males’ hypothalamus. It is important to highlight here that transcriptomic analysis in the present work allowed for the discovery of new players in the aromatase‐dependent neuroplasticity, but the functional role of these proteins and their causal link with local estrogen production remains to be investigated. However, this specific approach, while being extremely sensitive, is somewhat limited by the extreme heterogeneity of the brain tissue and is likely to lead to false negatives. For example, the increase of PCNA in the olfactory bulb and the concomitant reduction in the telencephalic region observed using immunofluorescence were not paralleled with transcriptomic analysis, likely due to the relatively low number of proliferating cells.

## 5 Conclusions

Our study revealed that brain aromatase influences adult behavior and neuroplasticity in zebrafish. Using a mutant model of the *cyp19a1b* gene, which encodes brain aromatase, we observed alterations in locomotion, aggressiveness, and sociability in mutant fish compared to wild‐type fish. In addition, changes in cell proliferation were detected in a sex‐ and brain region‐specific manner. Transcriptomic analysis nevertheless revealed that the mutation significantly affects various biological processes, including neurogenesis, behavior, the sensory system, estrogen signaling, cholesterol synthesis, and synaptic plasticity. These results offer new insights into the central role of aromatase/estrogen signaling in the regulation of neuroplasticity and behavior in zebrafish.

## Author Contributions

**Cassandra Malleret:** methodology, formal analysis, investigation, data curation, writing – original draft, visualization. **Mélanie Blanc‐Legendre:** methodology, formal analysis, data curation, investigation, writing – original draft, visualization. **Laëtitia Guillot:** methodology, formal analysis, investigation, writing – review and editing. **Harmony Lautrette‐Quinveros:** investigation. **Pavlina Pavlidi:** investigation. **Christina Dalla:** investigation. **Nikos Kokras:** investigation. **François Brion:** funding acquisition. **Maryne Toupin:** investigation. **Frédéric Chalmel:** methodology, software, formal analysis. **Xavier Cousin:** conceptualization, methodology, investigation, writing – review and editing. **Thierry Dominique Charlier:** conceptualization, methodology, investigation, writing – review and editing, supervision. **Elisabeth Pellegrini:** conceptualization, methodology, investigation, writing – review and editing, supervision.

## Ethics Statement

This work has been performed in agreement with Directive 2010/63/EU and received specific approval of the Ministry of Research under project authorization number APAFIS #32568‐2021072622109117 v3. Animal experiments were performed at MARBEC Palavas Experimental Marine Platform, which has agreement D34121926 for animal experiments.

## Conflicts of Interest

The authors declare no conflicts of interest.

## Peer Review

The peer review history for this article is available at https://www.webofscience.com/api/gateway/wos/peer‐review/10.1111/jnc.70202.

## Supporting information


**Figure S1:** Percentage of survival of zebrafish WT and mutant larvae every 24 hpf to 120 hpf on 40 (WT) and 44 (mutant) egg‐laying (A). Developmental parameters measured in embryos or larvae: Heartbeat frequency (beats per min) at 2 dpf; total body length at 5 dpf; size of swim bladder at 5 dpf; size of eye at 5 dpf (B). Length, weight, and sex ratio of adult zebrafish from mutant and WT line (C). Data are shown as Mean ± SD. **p < 0.01.

**Figure S2:** Results from reproductive behavior assessment: Distance traveled during reproduction test; average interindividual distance; time spent in the spawning area; time spent in body contact, for WT and mutant adult fish (A). Fertilization rate (B). Data are shown as mean ± SD. #*p* < 0.1 versus the WT group.

**Figure S3:** Time spent in the novel arm of the y‐maze and time spent in the top zone of the novel tank for WT and mutant adult fish. Data are shown as the mean ± SD. #*p* < 0.1 versus the WT group.

**Figure S4:** Quantification of the number of PCNA‐labeled cells in WT and mutant fish. (A) Dorsal telencephalon (D). (B) Thalamus. (C) Dorsal zone of periventricular hypothalamus (Hd). (D) Ventral zone of periventricular hypothalamus (Hv). (E) Pituitary. Red dots on schematic frontal sections indicate quantified areas. Mean ± SEM.

**Figure S5:** Zebrafish Cyp19a1b‐mutant vs. WT differentially expressed genes (DEGs) networked with Cytoscape software. Some of the DEGs are grouped by selected Gene Ontology Biological Process of interest (in circle). (A) DEGs in male olfactory bulbs. (B) DEGs in female olfactory bulbs. (C) DEGs in male telencephalon. (A') DEGs in female telencephalon. (B′) DEGs in male hypothalamus. (C′) DEGs in female hypothalamus.

**Table S1:** Primary and secondary antibodies.

**Table S2:** Summary of behavioral results obtained in the different behavioral tests (Mean ± SEM). Values significantly different from controls are in bold.

**Table S3:** Summary of the monoamine and metabolite levels in the brain of WT and mutant fish. NA, DA, 5‐HT, DOPAC, 5HIAA, HVA, and 3MT were expressed in ng per mg of protein, and the turnover was expressed as the ratio between metabolites and monoamines. Mean ± SEM, the number of analyzed brains is indicated in each cell. Values significantly different from controls are in bold.

**Table S4:** Excel file of significant differentially expressed genes in males vs. females and mutants vs. WT in the olfactory bulb, the telencephalon, and the hypothalamus.

## Data Availability

The data that support the findings of this study are available on request from the corresponding author. The data are not publicly available due to privacy or ethical restrictions.
