# Supplementary figures and images for: Mutation of Brain Aromatase Impairs Behavior and Neuroplasticity in Adult Zebrafish
Source: J Neurochem. 2025 Aug 25;169(8):e70202. doi: 10.1111/jnc.70202 (PMC12376962; doi:10.1111/jnc.70202)

A

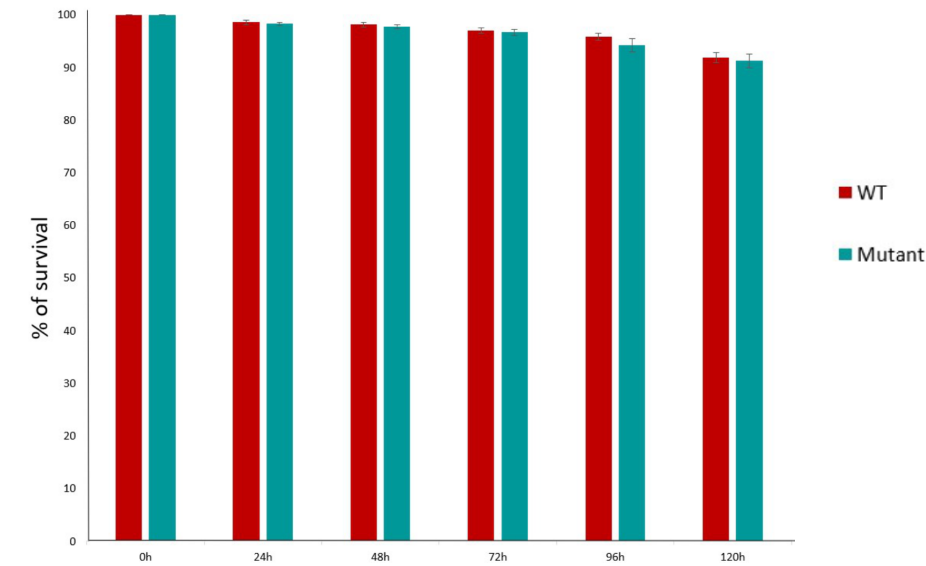

B

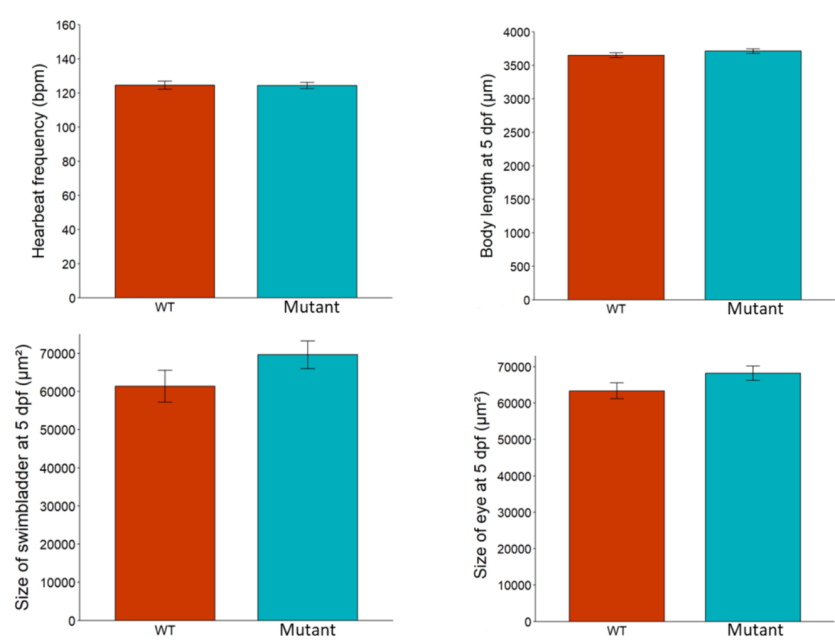

C

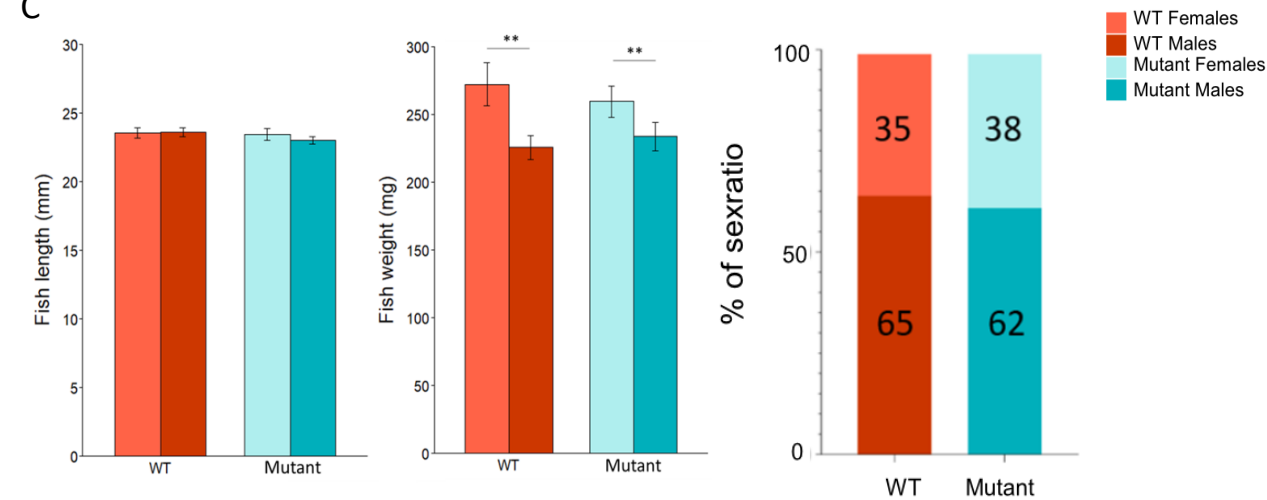

Supplement: Supplementary file 1 — Figure S1: Percentage of survival of zebrafish WT and mutant larvae every 24 hpf to 120 hpf on 40 (WT) and 44 (mutant) egg‐laying (A). Developmental parameters measured in embryos or larvae: Heartbeat frequency (beats per min) at 2 dpf; total body length at 5 dpf; size of swim bladder at 5 dpf; size of eye at 5 dpf (B). Length, weight, and sex ratio of adult zebrafish from mutant and WT line (C). Data are shown as Mean ± SD. **p < 0.01. [file JNC-169-0-s003.pdf]

A

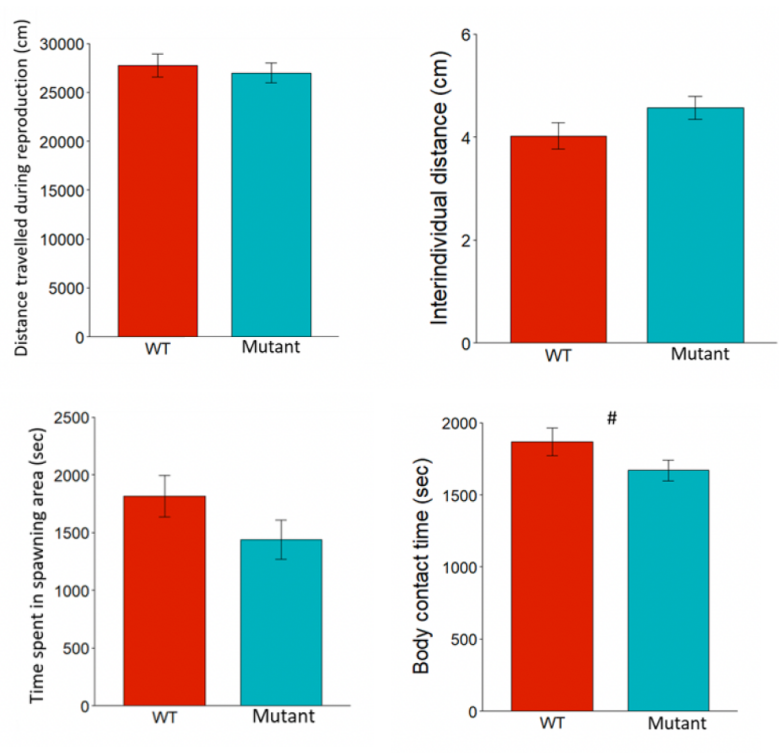

B

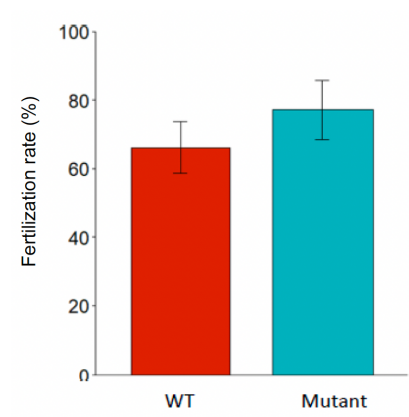

Supplement: Supplementary file 2 — Figure S2: Results from reproductive behavior assessment: Distance traveled during reproduction test; average interindividual distance; time spent in the spawning area; time spent in body contact, for WT and mutant adult fish (A). Fertilization rate (B). Data are shown as mean ± SD. #p < 0.1 versus the WT group. [file JNC-169-0-s004.pdf]

**A**

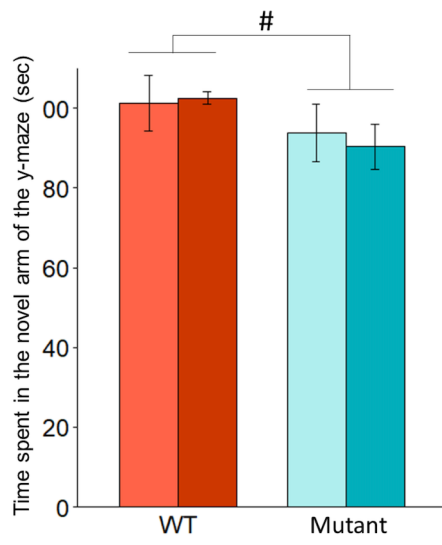

**B**

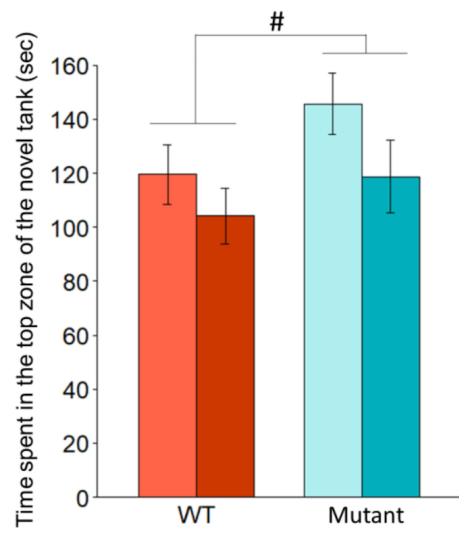

Supplement: Supplementary file 3 — Figure S3: Time spent in the novel arm of the y‐maze and time spent in the top zone of the novel tank for WT and mutant adult fish. Data are shown as the mean ± SD. #p < 0.1 versus the WT group. [file JNC-169-0-s001.pdf]

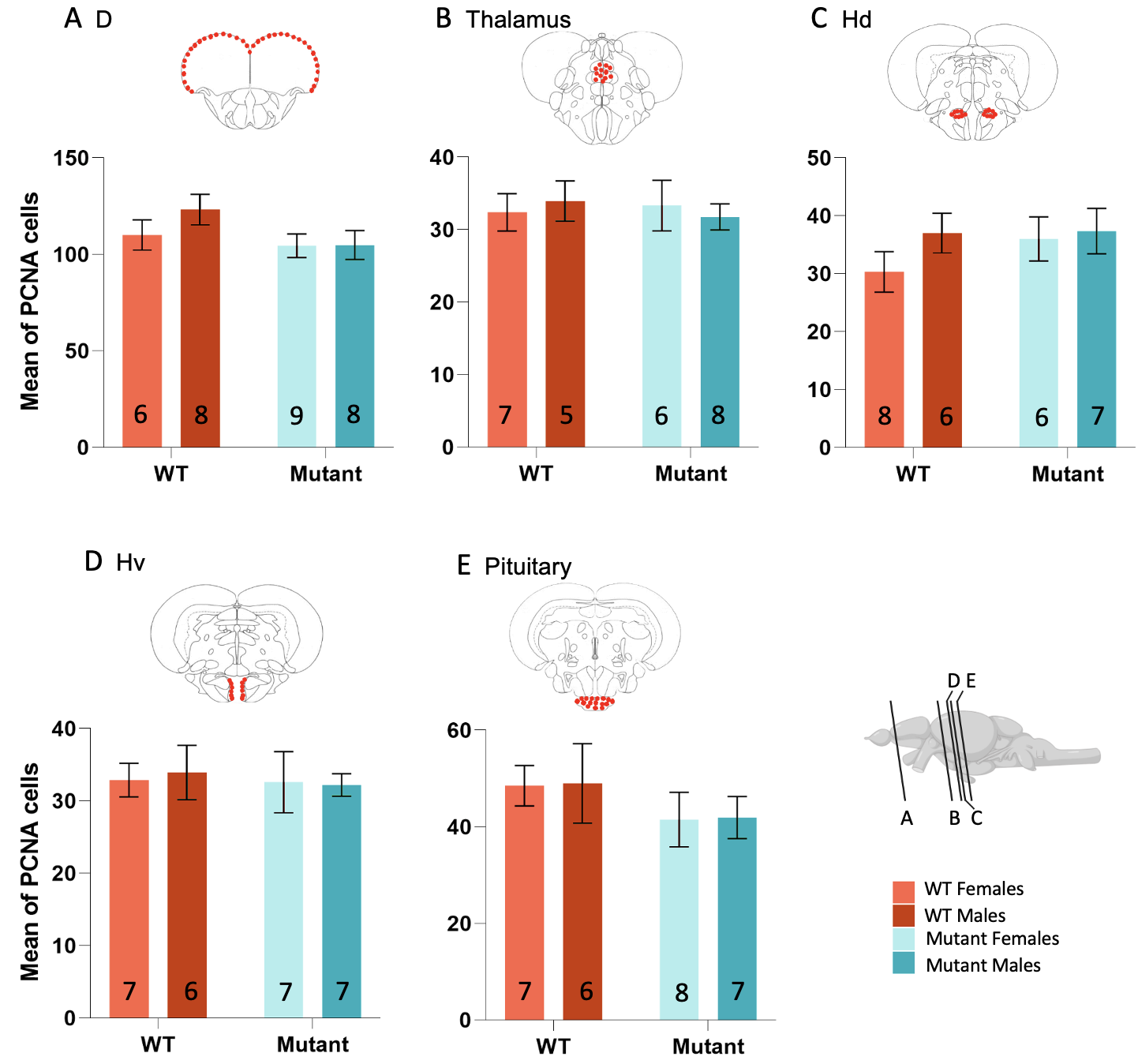

Supplement: Supplementary file 4 — Figure S4: Quantification of the number of PCNA‐labeled cells in WT and mutant fish. (A) Dorsal telencephalon (D). (B) Thalamus. (C) Dorsal zone of periventricular hypothalamus (Hd). (D) Ventral zone of periventricular hypothalamus (Hv). (E) Pituitary. Red dots on schematic frontal sections indicate quantified areas. Mean ± SEM. [file JNC-169-0-s007.png]
