# Supplementary material for: Mutation of Brain Aromatase Impairs Behavior and Neuroplasticity in Adult Zebrafish
Source: J Neurochem. 2025 Aug 25;169(8):e70202. doi: 10.1111/jnc.70202 (PMC12376962; doi:10.1111/jnc.70202)
Supplement: Supplementary file 5 — Figure S5: Zebrafish Cyp19a1b‐mutant vs. WT differentially expressed genes (DEGs) networked with Cytoscape software. Some of the DEGs are grouped by selected Gene Ontology Biological Process of interest (in circle). (A) DEGs in male olfactory bulbs. (B) DEGs in female olfactory bulbs. (C) DEGs in male telencephalon. (A') DEGs in female telencephalon. (B′) DEGs in male hypothalamus. (C′) DEGs in female hypothalamus. [file JNC-169-0-s005.pdf]

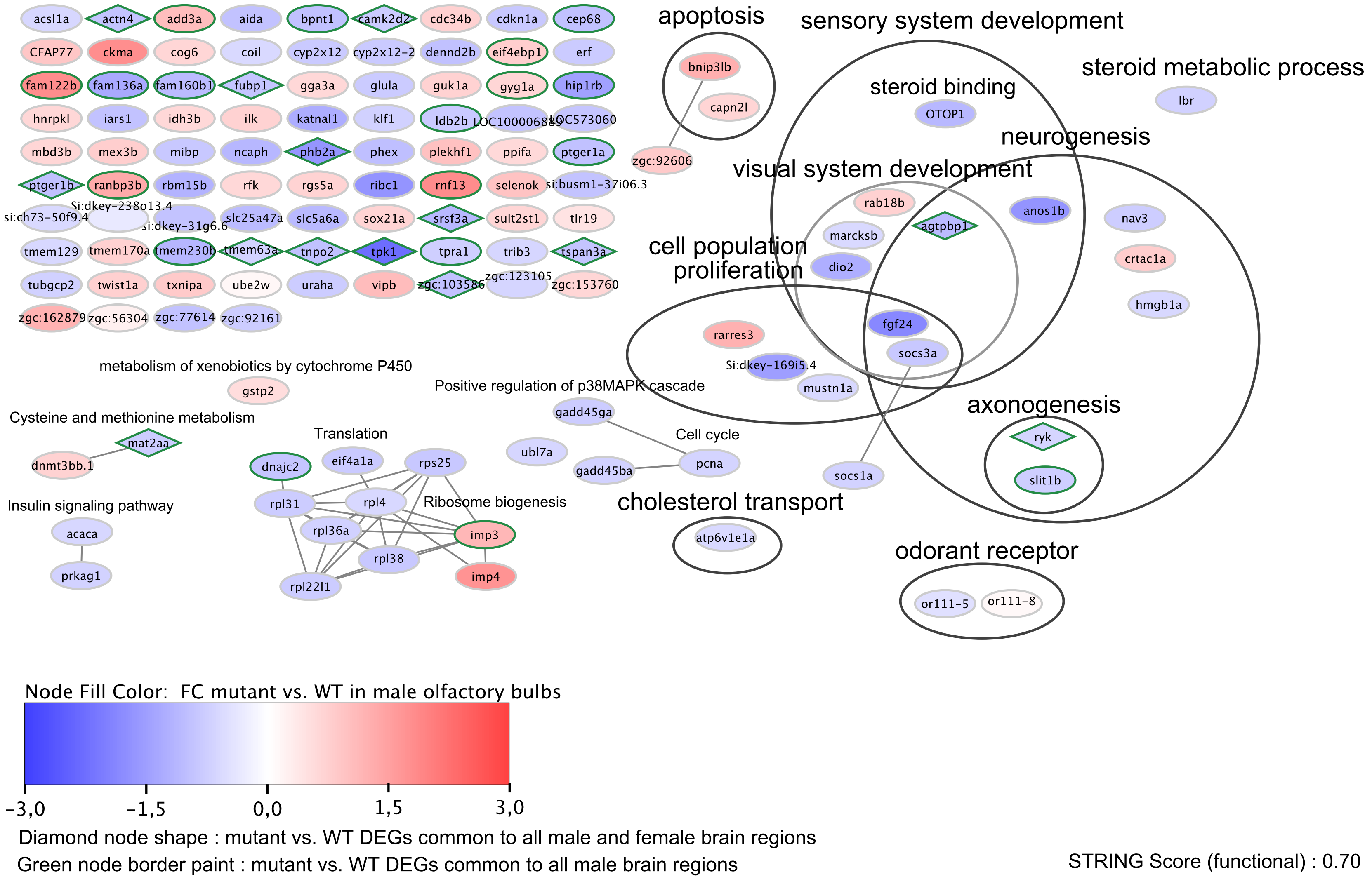

acsl1a
actn4
add3a
aida
bpnt1
camk2d2
cdc34b
cdkn1a
cep68
CFAP77
ckma
cog6
coil
cyp2x12
cyp2x12-2
dennd2b
eif4ebp1
erf
fam122b
fam136a
fam160b1
fubp1
gga3a
glula
guk1a
gyg1a
hip1rb
hnrpkl
iars1
idh3b
ilk
katnal1
klf1
ldb2b
mbd3b
mex3b
mibp
ncaph
phb2a
phex
plekhf1
ppifa
ptger1a
ptger1b
ranbp3b
rbm15b
rfk
rgs5a
ribc1
rnf13
selenok
si:busm1-37i06.3
Si:dkey-238o13.4
si:ch73-50f9.4
si:dkey-31g6.6
slc25a47a
slc5a6a
sox21a
srsf3a
sult2st1
tlr19
tmem129
tmem170a
tmem230b
tmem63a
tnpo2
tpk1
tpra1
trib3
tspan3a
tubgcp2
twist1a
txnipa
ube2w
uraha
vipb
zgc:103586
zgc:123105
zgc:153760
zgc:162879
zgc:56304
zgc:77614
zgc:92161
apoptosis
bnip3lb
capn2l
zgc:92606
sensory system development
steroid binding
OTOP1
steroid metabolic process
lbr
neurogenesis
visual system development
rab18b
marcksb
agtpbp1
anos1b
nav3
dio2
crtac1a
cell population proliferation
hmgb1a
fgf24
rarres3
socs3a
Si:dkey-169i5.4
mustn1a
axonogenesis
ryk
slit1b
socs1a
metabolism of xenobiotics by cytochrome P450
gstp2
Positive regulation of p38MAPK cascade
gadd45ga
Cysteine and methionine metabolism
mat2aa
dnmt3bb.1
Translation
dnajc2
eif4a1a
rps25
ubl7a
Cell cycle
gadd45ba
pcna
rpl31
rpl4
Ribosome biogenesis
rpl36a
imp3
rpl38
imp4
rpl22l1
Insulin signaling pathway
acaca
prkag1
cholesterol transport
atp6v1e1a
odorant receptor
or111-5
or111-8
Node Fill Color: FC mutant vs. WT in male olfactory bulbs
-3,0
-1,5
0,0
1,5
3,0
Diamond node shape : mutant vs. WT DEGs common to all male and female brain regions
Green node border paint : mutant vs. WT DEGs common to all male brain regions
STRING Score (functional) : 0.70

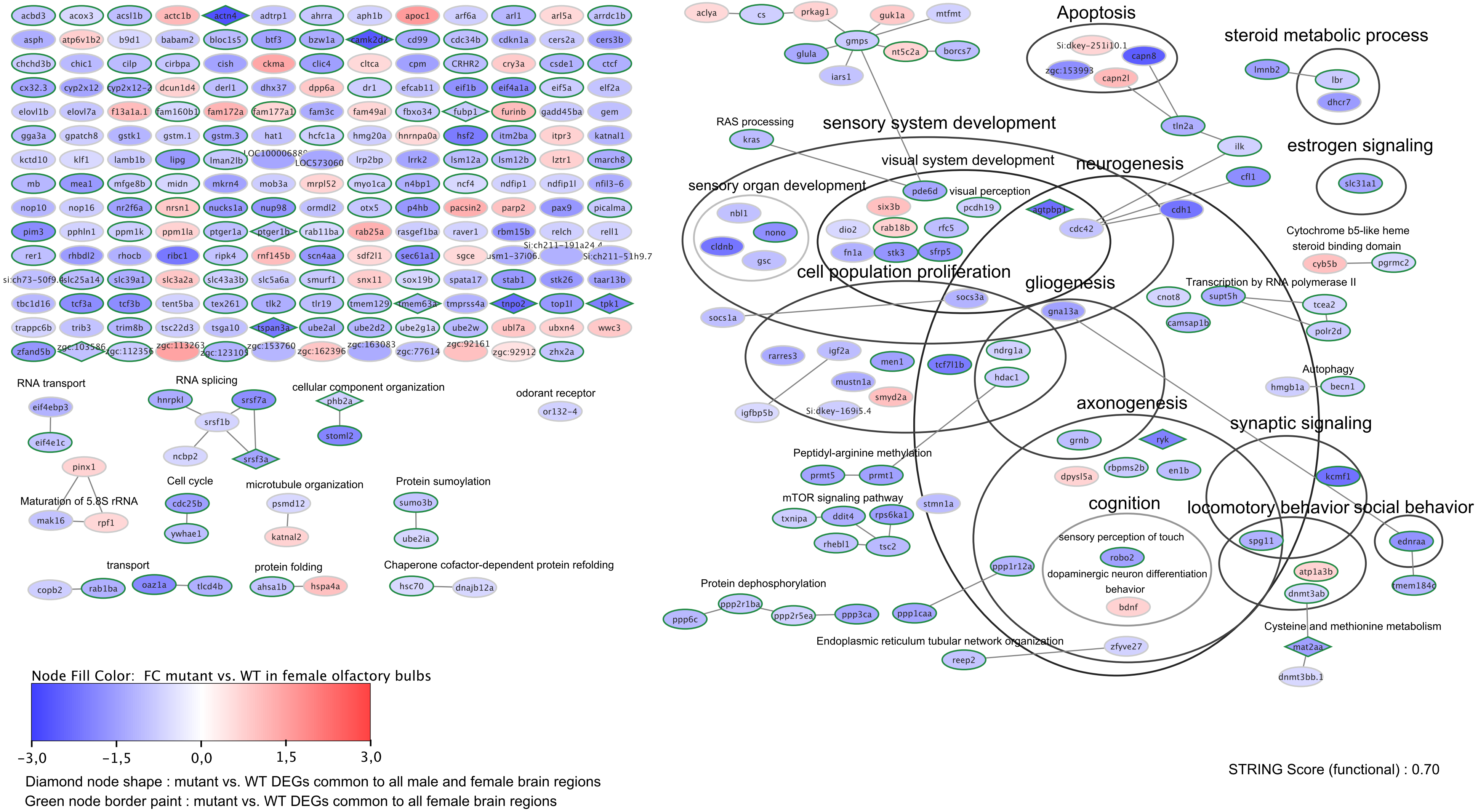

acbd3 acox3 acsl1b actc1b actn4 adtrp1 ahrra aph1b apoc1 arf6a arl1 arl5a arrdc1b
asph atp6v1b2 b9d1 babam2 bloc1s5 btf3 bzw1a camk2d2 cd99 cdc34b cdkn1a cers2a cers3b
chchd3b chic1 cilp cirbpa cish ckma clic4 cltca cpm CRHR2 cry3a csde1 ctcf
cx32.3 cyp2x12 cyp2x12-2 dcun1d4 derl1 dhx37 dpp6a dr1 efcab11 eif1b eif4a1a eif5a elf2a
elovl1b elovl7a f13a1a.1 fam160b1 fam172a fam177a1 fam3c fam49al fbxo34 fubp1 furinb gadd45ba gem
gga3a gpatch8 gstk1 gstm.1 gstm.3 hat1 hcfc1a hmg20a hnrnpa0a hsf2 itm2ba itpr3 katnal1
kctd10 klf1 lamb1b lipg lman2lb LOC573060 lrp2bp lrrk2 lsm12a lsm12b lztr1 march8
mb mea1 mfge8b midn mkrn4 mob3a mrpl52 myo1ca n4bp1 ncf4 ndfip1 ndfip1l nfil3-6
nop10 nop16 nr2f6a nrsn1 nucks1a nup98 ormdl2 otx5 p4hb pacsin2 parp2 pax9 picalma
pim3 pphln1 ppm1k ppm1la ptger1a ptger1b rab11ba rab25a rasgef1ba raver1 rbm15b relch rell1
rer1 rhbdl2 rhocb ribc1 ripk4 rnf145b scn4aa sdf2l1 sec61a1 sgce Si:ch211-191a24.4 Si:ch211-51h9.7
slc25a14 slc39a1 slc3a2a slc43a3b slc5a6a smurf1 snx11 sox19b spata17 stab1 stk26 taar13b
tbc1d16 tcf3a tcf3b tent5ba tex261 tlk2 tlr19 tmem129 tmem63a tmprss4a tnpo2 top1l tpk1
trappc6b trib3 trim8b tsc22d3 tsga10 tspan3a ube2al ube2d2 ube2g1a ube2w ubl7a ubxn4 wwc3
zfand5b zgc:103586 zgc:112356 zgc:113263 zgc:123105 zgc:153760 zgc:162396 zgc:163083 zgc:77614 zgc:92161 zgc:92912 zhx2a
RNA transport
eif4ebp3
eif4e1c
RNA splicing
hnrpkl
srsf7a
srsf1b
ncbp2
srsf3a
cellular component organization
phb2a
stoml2
odorant receptor
or132-4
pinx1
Maturation of 5.8S rRNA
mak16
rpf1
Cell cycle
cdc25b
ywhae1
microtubule organization
psmd12
katnal2
Protein sumoylation
sumo3b
ube2ia
transport
copb2
rab1ba
oaz1a
tlcd4b
protein folding
ahsa1b
hspa4a
Chaperone cofactor-dependent protein refolding
hsc70
dnajb12a
Node Fill Color: FC mutant vs. WT in female olfactory bulbs
-3,0
-1,5
0,0
1,5
3,0
Diamond node shape : mutant vs. WT DEGs common to all male and female brain regions
Green node border paint : mutant vs. WT DEGs common to all female brain regions
aclya
cs
prkag1
guk1a
mtfmt
gmps
glula
nt5c2a
borcs7
iars1
Apoptosis
Si:dkey-251i10.1
capn8
zgc:153993
capn2l
steroid metabolic process
lmnb2
lbr
dhcr7
tln2a
ilk
RAS processing
kras
sensory system development
visual system development
neurogenesis
estrogen signaling
cfl1
slc31a1
sensory organ development
pde6d
visual perception
nbl1
six3b
pcdh19
agtpbp1
cdh1
cldnb
nono
dio2
rab18b
rfc5
cdc42
gsc
fn1a
stk3
sfrp5
Cytochrome b5-like heme steroid binding domain
cyb5b
pgrmc2
cell population proliferation
gliogenesis
Transcription by RNA polymerase II
socs3a
cnot8
supt5h
tcea2
socs1a
gna13a
camsap1b
polr2d
rarres3
igf2a
men1
tcf7l1b
ndrg1a
hdac1
Autophagy
mustn1a
hmgb1a
becn1
smyd2a
igfbp5b
Si:dkey-169i5.4
axonogenesis
synaptic signaling
grnb
ryk
Peptidyl-arginine methylation
rbpms2b
en1b
prmt5
prmt1
dpysl5a
kcmf1
mTOR signaling pathway
stmn1a
cognition
locomotory behavior
social behavior
txnipa
ddit4
rps6ka1
sensory perception of touch
spg11
ednraa
rhebl1
tsc2
robo2
ppp1r12a
dopaminergic neuron differentiation
atp1a3b
behavior
tmem184c
Protein dephosphorylation
dnmt3ab
ppp2r1ba
bdnf
ppp6c
ppp2r5ea
ppp3ca
ppp1caa
Cysteine and methionine metabolism
Endoplasmic reticulum tubular network organization
zfyve27
mat2aa
reep2
dnmt3bb.1
STRING Score (functional) : 0.70

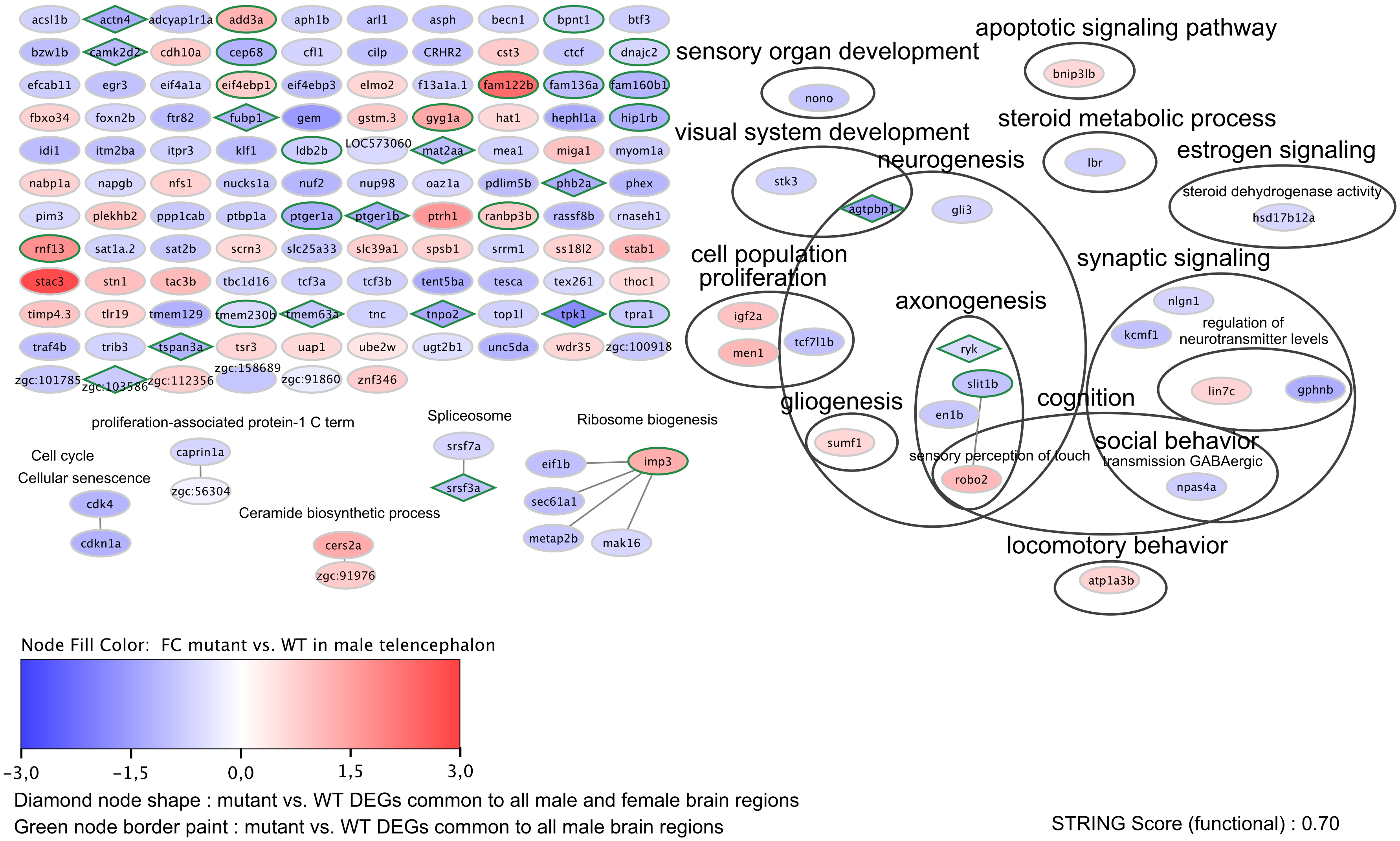

acsl1b actn4 adcyap1r1a add3a aph1b arl1 asph becn1 bpnt1 btf3
bzw1b camk2d2 cdh10a cep68 cfl1 cilp CRHR2 cst3 ctcf dnajc2
efcab11 egr3 eif4a1a eif4ebp1 eif4ebp3 elmo2 f13a1a.1 fam122b fam136a fam160b1
fbxo34 foxn2b ftr82 fubp1 gem gstm.3 gyg1a hat1 hephl1a hip1rb
idi1 itm2ba itpr3 klf1 ldb2b LOC573060 mat2aa mea1 miga1 myom1a
nabp1a napgb nfs1 nucks1a nuf2 nup98 oaz1a pdlim5b phb2a phex
pim3 plekhb2 ppp1cab ptbp1a ptger1a ptger1b ptrh1 ranbp3b rassf8b rnaseh1
rnf13 sat1a.2 sat2b scrn3 slc25a33 slc39a1 spsb1 srrm1 ss18l2 stab1
stac3 stn1 tac3b tbc1d16 tcf3a tcf3b tent5ba tesca tex261 thoc1
timp4.3 tlr19 tmem129 tmem230b tmem63a tnc tnpo2 top1l tpk1 tpra1
traf4b trib3 tspan3a tsr3 uap1 ube2w ugt2b1 unc5da wdr35 zgc:100918
zgc:101785 zgc:103586 zgc:112356 zgc:158689 zgc:91860 znf346
proliferation-associated protein-1 C term
caprin1a
zgc:56304
Cell cycle
Cellular senescence
cdk4
cdkn1a
Ceramide biosynthetic process
cers2a
zgc:91976
Spliceosome
srsf7a
srsf3a
Ribosome biogenesis
eif1b
imp3
sec61a1
metap2b
mak16
sensory organ development
nono
apoptotic signaling pathway
bnip3lb
steroid metabolic process
lbr
visual system development
stk3
neurogenesis
agtpbp1
gli3
estrogen signaling
steroid dehydrogenase activity
hsd17b12a
cell population proliferation
igf2a
men1
tcf7l1b
axonogenesis
ryk
slit1b
en1b
gliogenesis
sumf1
sensory perception of touch
robo2
synaptic signaling
nlgn1
kcmf1
regulation of neurotransmitter levels
lin7c
gphnb
cognition
social behavior
transmission GABAergic
npas4a
locomotory behavior
atp1a3b
Node Fill Color: FC mutant vs. WT in male telencephalon
−3,0 −1,5 0,0 1,5 3,0
Diamond node shape : mutant vs. WT DEGs common to all male and female brain regions
Green node border paint : mutant vs. WT DEGs common to all male brain regions
STRING Score (functional) : 0.70

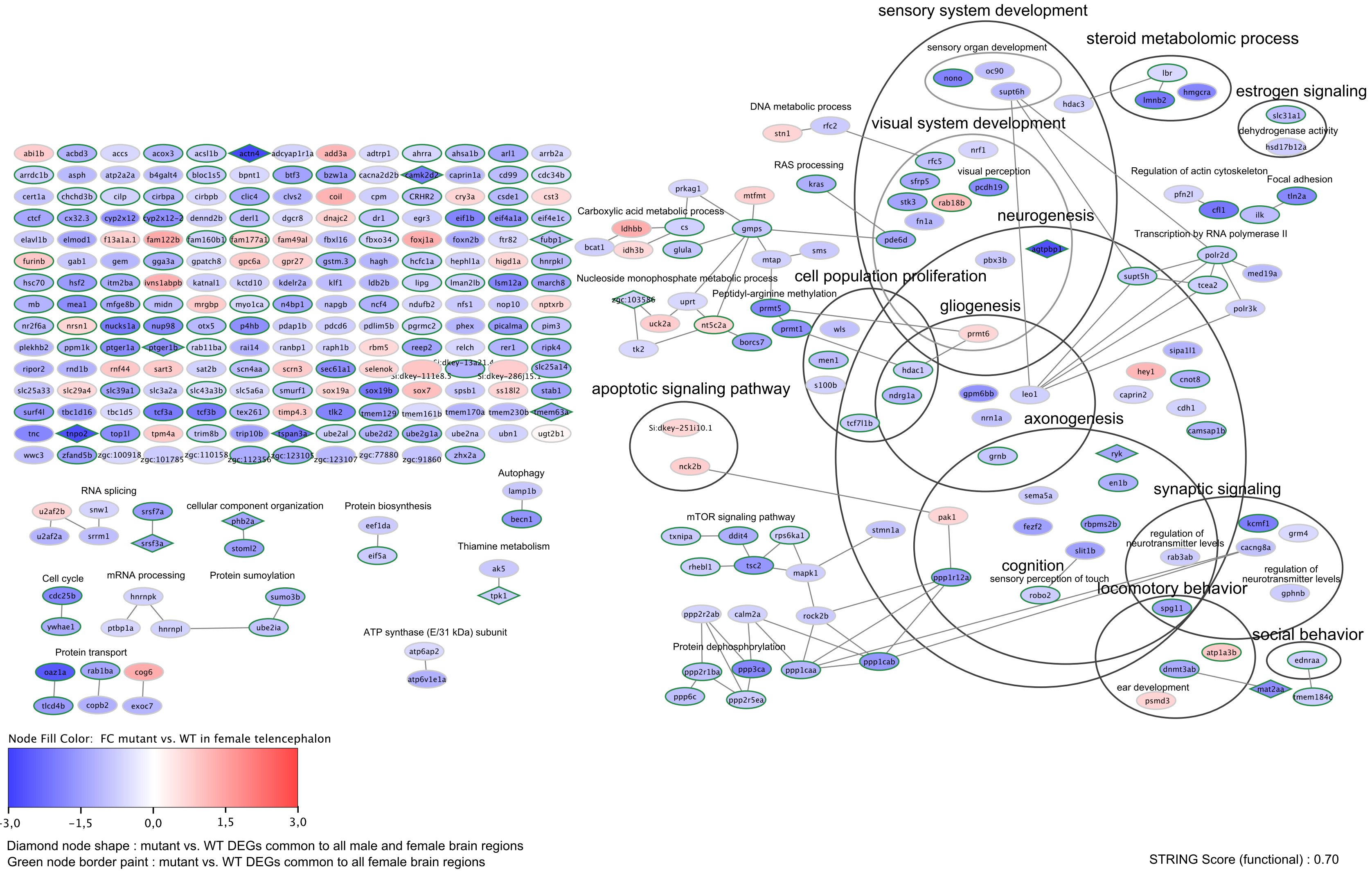

abi1b acbd3 accs acox3 acsl1b actn4 adcyap1r1a add3a adtrp1 ahrra ahsa1b arl1 arrb2a
arrdc1b asph atp2a2a b4galt4 bloc1s5 bpnt1 btf3 bzw1a cacna2d2b camk2d2 caprin1a cd99 cdc34b
cert1a chchd3b cilp cirbpa cirbpb clic4 clvs2 coil cpm CRHR2 cry3a csde1 cst3
ctcf cx32.3 cyp2x12 cyp2x12-2 dennd2b derl1 dgcr8 dnajc2 dr1 egr3 eif1b eif4a1a eif4e1c
elavl1b elmod1 f13a1a.1 fam122b fam160b1 fam177a1 fam49al fbxl16 fbxo34 foxj1a foxn2b ftr82 fubp1
furinb gab1 gem gga3a gpatch8 gpc6a gpr27 gstm.3 hagh hcfc1a hephl1a higd1a hnrpkl
hsc70 hsf2 itm2ba ivns1abpb katnal1 kctd10 kdelr2a klf1 ldb2b lipg lman2lb lsm12a march8
mb mea1 mfge8b midn mrgbp myo1ca n4bp1 napgb ncf4 ndufb2 nfs1 nop10 nptxrb
nr2f6a nrsn1 nucks1a nup98 otx5 p4hb pdap1b pdcd6 pdlim5b pgrmc2 phex picalma pim3
plekhb2 ppm1k ptger1a ptger1b rab11ba rai14 ranbp1 raph1b rbm5 reep2 relch rer1 ripk4
ripor2 rnd1b rnf44 sart3 sat2b scn4aa scrn3 sec61a1 selenok si:dkey-111e8.5 si:dkey-13a21.4 si:dkey-286j15.1 slc25a14
slc25a33 slc29a4 slc39a1 slc3a2a slc43a3b slc5a6a smurf1 sox19a sox19b sox7 spsb1 ss18l2 stab1
surf4l tbc1d16 tbc1d5 tcf3a tcf3b tex261 timp4.3 tlk2 tmem129 tmem161b tmem170a tmem230b tmem63a
tnc tnpo2 top1l tpm4a trim8b trip10b tspan3a ube2al ube2d2 ube2g1a ube2na ubn1 ugt2b1
wwc3 zfand5b zgc:100918 zgc:101785 zgc:110158 zgc:112356 zgc:123105 zgc:123107 zgc:77880 zgc:91860 zhx2a
RNA splicing
u2af2b snw1 srsf7a u2af2a srrm1 srsf3a
cellular component organization
phb2a stoml2
Protein biosynthesis
eef1da eif5a
Autophagy
lamp1b becn1
Thiamine metabolism
ak5 tpk1
Cell cycle
cdc25b ywhae1
mRNA processing
hnrnpk ptbp1a hnrnpl
Protein sumoylation
sumo3b ube2ia
Protein transport
oaz1a rab1ba cog6 tlcd4b copb2 exoc7
ATP synthase (E/31 kDa) subunit
atp6ap2 atp6v1e1a
Node Fill Color: FC mutant vs. WT in female telencephalon
−3,0 −1,5 0,0 1,5 3,0
Diamond node shape : mutant vs. WT DEGs common to all male and female brain regions
Green node border paint : mutant vs. WT DEGs common to all female brain regions
DNA metabolic process
stn1 rfc2
RAS processing
kras
Carboxylic acid metabolic process
prkag1 mtfmt ldhbb cs gmps bcat1 idh3b glula mtap sms
Nucleoside monophosphate metabolic process
zgc:103586 uprt uck2a nt5c2a tk2
Peptidyl-arginine methylation
prmt5 prmt1 borcs7
apoptotic signaling pathway
Si:dkey-251i10.1 nck2b
mTOR signaling pathway
txnipa ddit4 rps6ka1 rhebl1 tsc2 mapk1 stmn1a
ppp2r2ab calm2a rock2b
Protein dephosphorylation
ppp2r1ba ppp3ca ppp1caa ppp1cab ppp6c ppp2r5ea
sensory system development
sensory organ development
nono oc90 supt6h hdac3
visual system development
nrf1 rfc5 sfrp5 stk3 rab18b fn1a pde6d
visual perception
pcdh19
steroid metabolomic process
lbr lmnb2 hmgcra
estrogen signaling
slc31a1
dehydrogenase activity
hsd17b12a
Regulation of actin cytoskeleton
pfn2l cfl1 ilk
Focal adhesion
tln2a
neurogenesis
pbx3b agtpbp1
Transcription by RNA polymerase II
polr2d supt5h tcea2 med19a polr3k
cell population proliferation
wls men1 s100b tcf7l1b
gliogenesis
prmt6 hdac1 ndrg1a gpm6bb leo1 nrn1a
sipa1l1 hey1 cnot8 caprin2 cdh1 camsap1b
axonogenesis
grnb ryk sema5a en1b pak1 fezf2 rbpms2b slit1b ppp1r12a
cognition
sensory perception of touch
robo2
synaptic signaling
kcmf1 grm4
regulation of neurotransmitter levels
cacng8a rab3ab
regulation of neurotransmitter levels
gphnb
locomotory behavior
spg11 atp1a3b dnmt3ab
ear development
psmd3
social behavior
ednraa mat2aa tmem184c
STRING Score (functional) : 0.70

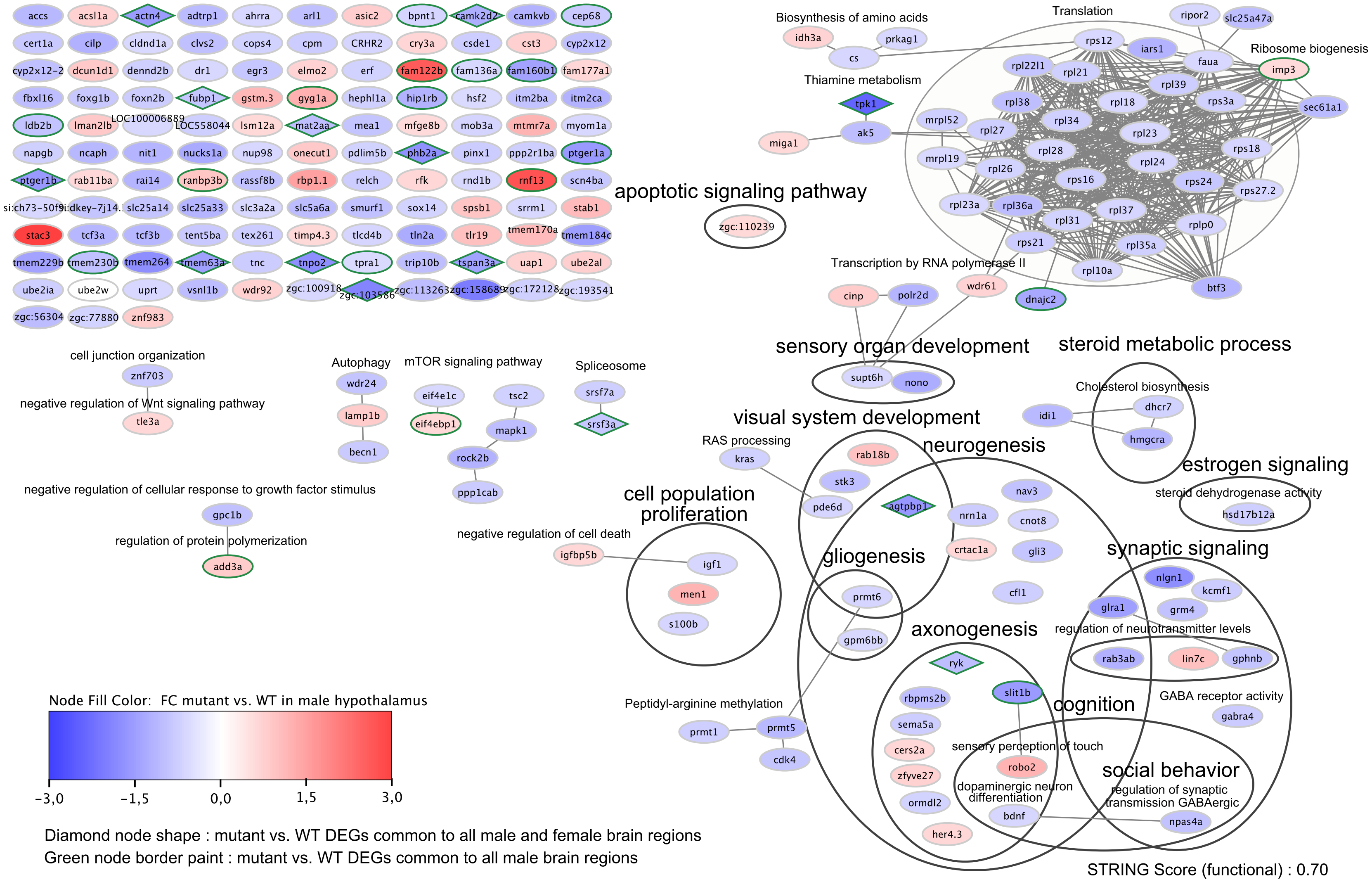

accs acsl1a actn4 adtrp1 ahrra arl1 asic2 bpnt1 camk2d2 camkvb cep68
cert1a cilp cldnd1a clvs2 cops4 cpm CRHR2 cry3a csde1 cst3 cyp2x12
cyp2x12-2 dcun1d1 dennd2b dr1 egr3 elmo2 erf fam122b fam136a fam160b1 fam177a1
fbxl16 foxg1b foxn2b fubp1 gstm.3 gyg1a hephl1a hip1rb hsf2 itm2ba itm2ca
ldb2b lman2lb LOC100006889 LOC558044 lsm12a mat2aa mea1 mfge8b mob3a mtmr7a myom1a
napgb ncaph nit1 nucks1a nup98 onecut1 pdlim5b phb2a pinx1 ppp2r1ba ptger1a
ptger1b rab11ba rai14 ranbp3b rassf8b rbp1.1 relch rfk rnd1b rnf13 scn4ba
si:ch73-50f9 si:dkey-7j14 slc25a14 slc25a33 slc3a2a slc5a6a smurf1 sox14 spsb1 srrm1 stab1
stac3 tcf3a tcf3b tent5ba tex261 timp4.3 tlcd4b tln2a tlr19 tmem170a tmem184c
tmem229b tmem230b tmem264 tmem63a tnc tnpo2 tpra1 trip10b tspan3a uap1 ube2al
ube2ia ube2w uprt vsnl1b wdr92 zgc:100918 zgc:103586 zgc:113263 zgc:158689 zgc:172128 zgc:193541
zgc:56304 zgc:77880 znf983
Biosynthesis of amino acids
idh3a prkag1 cs
Thiamine metabolism
tpk1 ak5 miga1
Translation
ripor2 slc25a47a
Ribosome biogenesis
rps12 iars1 faua imp3 rpl22l1 rpl21 rpl39 sec61a1 rpl38 rpl18 rps3a mrpl52 rpl34 rpl27 rpl23 rps18 mrpl19 rpl28 rpl24 rpl26 rps16 rps24 rps27.2 rpl23a rpl36a rpl31 rpl37 rplp0 rps21 rpl35a rpl10a btf3
apoptotic signaling pathway
zgc:110239
Transcription by RNA polymerase II
cinp polr2d wdr61 dnajc2
sensory organ development
supt6h nono
steroid metabolic process
Cholesterol biosynthesis
idi1 dhcr7 hmgcra
estrogen signaling
steroid dehydrogenase activity
hsd17b12a
visual system development
neurogenesis
RAS processing
kras
rab18b stk3 pde6d agtpbp1 nrn1a crtac1a nav3 cnot8 gli3 cfl1
gliogenesis
prmt6 gpm6bb
cell population proliferation
negative regulation of cell death
igfbp5b igf1 men1 s100b
synaptic signaling
nlgn1 kcmf1 glra1 grm4
regulation of neurotransmitter levels
rab3ab lin7c gphnb
axonogenesis
ryk slit1b rbpms2b sema5a cers2a zfyve27 ormdl2 her4.3
cognition
GABA receptor activity
gabra4
sensory perception of touch
robo2
dopaminergic neuron differentiation
bdnf
social behavior
regulation of synaptic transmission GABAergic
npas4a
Peptidyl-arginine methylation
prmt1 prmt5 cdk4
cell junction organization
znf703
negative regulation of Wnt signaling pathway
tle3a
Autophagy
wdr24 lamp1b becn1
mTOR signaling pathway
eif4e1c eif4ebp1 tsc2 mapk1 rock2b ppp1cab
Spliceosome
srsf7a srsf3a
negative regulation of cellular response to growth factor stimulus
gpc1b
regulation of protein polymerization
add3a
Node Fill Color: FC mutant vs. WT in male hypothalamus
−3,0 −1,5 0,0 1,5 3,0
Diamond node shape : mutant vs. WT DEGs common to all male and female brain regions
Green node border paint : mutant vs. WT DEGs common to all male brain regions
STRING Score (functional) : 0.70

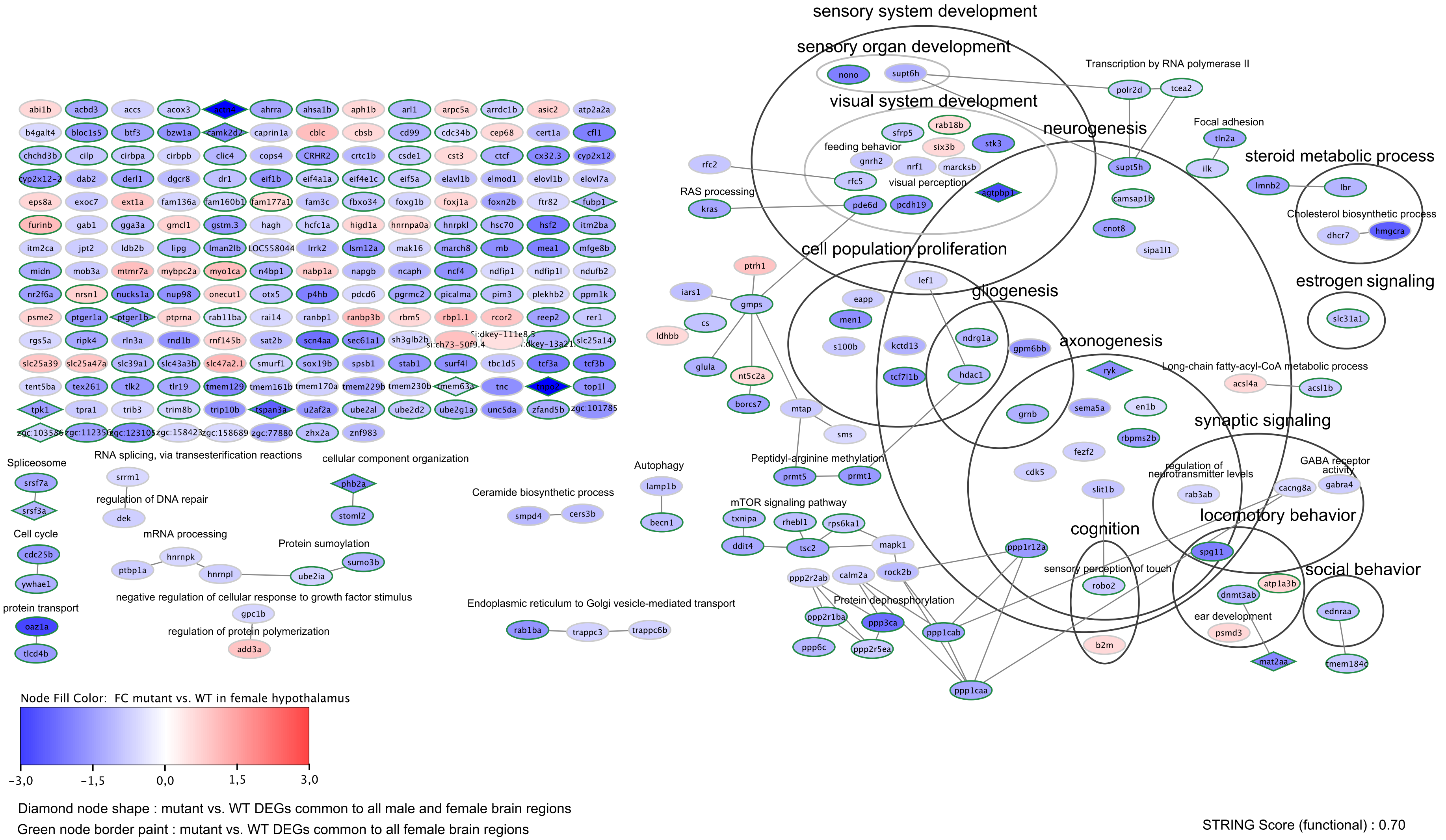

sensory system development
sensory organ development
visual system development
feeding behavior
visual perception
neurogenesis
Transcription by RNA polymerase II
Focal adhesion
steroid metabolic process
Cholesterol biosynthetic process
RAS processing
cell population proliferation
gliogenesis
axonogenesis
estrogen signaling
Long-chain fatty-acyl-CoA metabolic process
synaptic signaling
Peptidyl-arginine methylation
regulation of neurotransmitter levels
GABA receptor activity
mTOR signaling pathway
cognition
locomotory behavior
sensory perception of touch
social behavior
Protein dephosphorylation
ear development
Spliceosome
RNA splicing, via transesterification reactions
cellular component organization
Autophagy
regulation of DNA repair
Ceramide biosynthetic process
Cell cycle
mRNA processing
Protein sumoylation
negative regulation of cellular response to growth factor stimulus
protein transport
regulation of protein polymerization
Endoplasmic reticulum to Golgi vesicle-mediated transport
Node Fill Color: FC mutant vs. WT in female hypothalamus
−3,0
−1,5
0,0
1,5
3,0
Diamond node shape : mutant vs. WT DEGs common to all male and female brain regions
Green node border paint : mutant vs. WT DEGs common to all female brain regions
STRING Score (functional) : 0.70
