# Supplementary material for: Mutation of Brain Aromatase Impairs Behavior and Neuroplasticity in Adult Zebrafish
Source: J Neurochem. 2025 Aug 25;169(8):e70202. doi: 10.1111/jnc.70202 (PMC12376962; doi:10.1111/jnc.70202)
Supplement: Supplementary file 6 — Table S1: Primary and secondary antibodies. [file JNC-169-0-s002.docx]

Table S1: Primary and secondary antibodies

| Primary Antibodies | | | | |  |
| --- | --- | --- | --- | --- | --- |
| Antigen | Host | Dilution | Company | Cat. no. | RRID |
| AroB | Rabbit (polyclonal) | 1 :500 | Dr. François Brion  INERIS, France |  |  |
| PCNA clone PC10 | Mouse (monoclonal) | 1 :100 | DAKO | MO879 |  |
| TH | Rabbit (polyclonal) | 1 :500 | Merck Millipore | AB152 | AB_390204 |
| 5-HT | Rabbit (polyclonal) | 1 :4000 | Dr. Yves Tillet  INRA, France |  |  |

| **Secondary Antibodies** | | | | | | |
| --- | --- | --- | --- | --- | --- | --- |
| Antigen | Fluorophore | Host | Dilution | Company | Cat. no. | RRID |
| Mouse IgG | TRITC | Goat | 1:500 | ThermoFisher | A11032 | AB_2534091 |
| Rabbit IgG | FITC | Goat | 1:500 | ThermoFisher | A11034 | AB_2576217 |
