# Supplementary material for: Mutation of Brain Aromatase Impairs Behavior and Neuroplasticity in Adult Zebrafish
Source: J Neurochem. 2025 Aug 25;169(8):e70202. doi: 10.1111/jnc.70202 (PMC12376962; doi:10.1111/jnc.70202)
Supplement: Supplementary file 7 — Table S2: Summary of behavioral results obtained in the different behavioral tests (Mean ± SEM). Values significantly different from controls are in bold. [file JNC-169-0-s008.docx]

Table S2 : Summary of behavioral results obtained in the different behavioral tests (Mean±SEM). Values significantly different from controls are in bold.

| **Variable** | **WT** | | **Mutant** | |
| --- | --- | --- | --- | --- |
|  | **F** | **M** | **F** | **M** |
| **Shoaling test** |  | | | |
| Interindividual distance (mm)  Genotype effect:  t_20_=-1.55; p=0.138 | 137±22 | | 121±28 | |
| Distance traveled (mm)  Genotype effect:  t_122_=4.46; **p=0.0000018** | 32484±6216 | | **37190±5810** | |
| **Y-maze** |  | | | |
| Distance traveled (mm)  Genotype effect:  F_1,46_= 9.9810; **p=0.0027**  Sex effect:  F_1,46_= 1.020; p=0.32  Genotype*Sex effect:  F_1,46_= 0.011; p=0.92 | 1802±94 | 1914±136 | **1492±89** | **1583±77** |
| Alternations between arms  Genotype effect:  F_1,46_= 0.67; p=0.42  Sex effect:  (F_1,46_= 7.88; **p=0.0073**)  Genotype*Sex effect:  F_1,46_= 0.034; p=0.85 | 48±2 | 63±7 | 52±5 | 65±5 |
| Time spent in novel arm (sec)  Genotype effect:  F_1,46_=2.84; p=0.099  Sex effect:  F_1,46_= 0.033; p=0.86  Genotype*Sex effect:  F_1,46_= 0.16; p=0.69 | 101±7 | 103±2 | 94±7 | 90±6 |
| **Classical conditioning** |  | | | |
| Time spent out of the checkboard area (sec)  Genotype effect:  F_1,46_= 0.88; p=0.35  Sex effect:  F_1,46_= 1.94; p=0.17  Genotype*Sex effect:  F_1,46_= 0.33; p=0.97 | 85±1 | 76±5 | 78±5 | 76±4 |
| Visits to checkboard area  Genotype effect:  F_1,46_= 0.55; p=0.46  Sex effect:  F_1,46_= 0.51; p=0.48  Genotype*Sex effect:  F_1,46_ = 6.19; **p=0.017** | 5±1 | 4±1 | 3±1 | 5±1 |
| Distance traveled (mm)  Genotype effect:  F_1,46_= 0.47; p=0.50  Sex effect:  F_1,46_=3.34; p=0.074  Genotype*Sex effect:  (F_1,46_= 0.055; p=0.81) | 65035±4258 | 71801±5207 | 60807±1879 | 69575±4744 |
| **Z-maze** |  | | | |
| Distance travelled  Genotype effect:  F_1,46_= 0.34; p=0.56  Sex effect:  F_1,46_= 22.42; **p=0.000021**  Genotype*Sex effect:  F_1,46_= 2.27; p=0.14 | 25479±1355 | 30030±1329 | 22268±1537 | 31072±1412 |
| Latency to reach zone 16 (sec)  Genotype effect:  F_1,46_= 0.0082; p=0.93  Sex effect:  F_1,46_=13.55 ; **p=0.00061**  Genotype*Sex effect  F_1,46_= 0.025; p=0.87 | 371±67 | 180±8 | 375±73 | 199±12 |
| Latency to reach distal zone (sec)  Genotype effect:  F_1,46_= 0.13; p=0.72  Sex effect  F_1,46_= 6.04; **p=0.018**  Genotype*Sex effect  F_1,46_= 0.15; p=0.70 | 275±55 | 160±27 | 249±59 | 165±6 |
| Time spent in distal zone (sec)  Genotype effect:  F_1,46_= 0.39; p=0.53  Sex effect:  F_1,46_=5.63; **p=0.022**  Genotype*Sex effect:  F_1,46_= 0.23; p=0.63 | 81±16 | 117±10 | 94±14 | 118±9 |
| Time to exit shelter (sec)  Genotype effect  F_1,46_= 6.28; **p=0.016**  Sex effect:  F_1,46_= 6.01; **p=0.018**  Genotype*Sex effect  (F_1,46_= 2.02; p=0.16) | 139±3 | 131±2 | **131±2** | **129±1** |
| Time spent in proximal zone (sec)  Genotype effect:  F_1,46_= 1.78; p=0.19  Sex effect:  F_1,46_=11.19; **p=0.0016**  Genotype*Sex effect:  F_1,46_= 0.90; p=0.35 | 183±16 | 132±9 | 158±13 | 129±8 |
| **Novel tank diving test** |  | | | |
| Distance travelled (mm)  Genotype effect:  F_1,46_= 9.10; **p=0.0042**  Sex effect  F_1,46_= 6.89; **p=0.012**  Genotype*Sex effect:  (F_1,46_= 0.0060; p=0.94) | 12681±800 | 14775±1116 | **10323±542** | **12298±518** |
| Visits to top zone  Genotype effect:  (F_1,46_= 0.47; p=0.50)  Sex effect:  F_1,46_= 6.83; **p=0.012**  Genotype*Sex effect:  F_1,46_= 0.000; p=1.0 | 26±4 | 37±5 | 23±3 | 34±4 |
| Time spent in top zone (sec)  Genotype effect:  F_1,46_= 2.79; p=0.10  Sex effect:  F_1,46_= 3.30; p=0.076  Genotype*Sex effect:  F_1,46_= 0.24; p=0.62 | 120±11 | 104±10 | 146±11 | 119±13 |
| **Sociability** |  | | | |
| Distance travelled (mm)  Genotype effect:  F_1,46_= 0.24 ; p=0.63  Sex effect:  F_1,46_= 5.34; **p=0.025**  Genotype*Sex effect:  F_1,46_= 0.051; p=0.82 | 8849±315 | 10092±439 | 9154±524 | 10175±624 |
| Visits to social area  Genotype effect:  F_1,46_= 4.80; **p=0.034**  Sex effect:  F_1,46_=3.35 ; p=0.073  Genotype*Sex effect:  F_1,46_=4.42 ; **p=0.041** | 24±4 | 23±4 | 24±5 | **41±4** |
| Time spent in social area (sec)  Genotype effect:  F_1,46_= 1.92; p=0.17  Sex effect:  F_1,46_= 0.0007; p=0.98  Genotype*Sex effect:  F_1,46_= 0.091; p=0.76 | 255±14 | 249±14 | 226±26 | 231±14 |
| **Aggressiveness** |  | | | |
| Distance travelled (mm)  Genotype effect:  F_1,46_= 1.33; p=0.25  Sex effect:  F_1,46_= 18.50; **p=0.000088**  Genotype*Sex effect:  F_1,46_= 0.096; p=0.76 | 10376±249 | 12802±820 | 10784±269 | 13586±817 |
| Visits to mirror zone  Genotype effect:  F_1,46_= 8.03; **p=0.0068**  Sex effect:  F_1,46_= 18.97; **p=0.000073**  Genotype*Sex effect:  F_1,46_=2.90 ; p=0.095 | 43±6 | 99±16 | **31±4** | **56±7** |
| Time spent in mirror zone (sec)  Genotype effect:  F_1,46_=2.98 ; p=0.091  Sex effect:  F_1,46_= 17.93; **p=0.00011**  Genotype*Sex effect:  F_1,46_= 0.52; p=0.47 | 52±10 | 135±22 | 32±8 | 91±21 |
