# Supplementary material for: Mutation of Brain Aromatase Impairs Behavior and Neuroplasticity in Adult Zebrafish
Source: J Neurochem. 2025 Aug 25;169(8):e70202. doi: 10.1111/jnc.70202 (PMC12376962; doi:10.1111/jnc.70202)
Supplement: Supplementary file 8 — Table S3: Summary of the monoamine and metabolite levels in the brain of WT and mutant fish. NA, DA, 5‐HT, DOPAC, 5HIAA, HVA, and 3MT were expressed in ng per mg of protein, and the turnover was expressed as the ratio between metabolites and monoamines. Mean ± SEM, the number of analyzed brains is indicated in each cell. Values significantly different from controls are in bold. [file JNC-169-0-s009.docx]

Table S3: Summary of the monoamines and metabolites levels in the brain of WT and mutant fish. NA, DA, 5-HT, DOPAC, 5HIAA, HVA and 3MT were expressed in ng per mg of protein, and the turnover expressed as the ratio between metabolites and monoamines. Mean±SEM, the number of analyzed brains is indicated in each cell. Values significantly different from controls are in bold.

|  | **WT** | | **Mutant** | |
| --- | --- | --- | --- | --- |
|  | **Female** | **Male** | **Female** | **Male** |
| **Noradrenaline (NA)**  Genotype effect: F_(1,34)_=2.17366; p=0.14039  Sex effect:  F_(1,34)_=0.14444; p=0.7039  Interaction genotype*sex:  F_(1,34)_=0.00398; p=0.9497 | 11.44±1.90  N=9 | 11.77±1.95  N=10 | 14.17±1.80  N=9 | 14.64±1.88  N=10 |
| **Dopamine (DA)**  Genotype effect: F_(1,34)_=1.32985; p=0.24883  Sex effect:  F_(1,34)_=0.27692; p=0.59873  Interaction genotype*sex:  F_(1,34)_=0.07842; p=0.77944 | 2.24±0.45  N=9 | 2.41±0.53  N=10 | 1.82±0.45  N=9 | 2.22±0.43  N=10 |
| **Serotonin (5-HT)**  Genotype effect: F_(1,34)_=0.3222; p=0.5740  Sex effect:  F_(1,34)_=0.5442; p=0.4658  Interaction genotype*sex:  F_(1,34)_=0.05132; p=0.8221 | 0.70±0.14  N=9 | 0.85±0.18  N=10 | 0.64±0.17  N=9 | 0.73±0.15  N=10 |
| **DOPAC**  Genotype effect: F_(1,34)_=0.15534; p=0.69349  Sex effect:  F_(1,34)_=0.67009; p=0.41302  Interaction genotype*sex:  F_(1,34)_=0.21308; p=0.64436 | 0.36±0.05  N=9 | 0.43±0.08  N=10 | 0.40±0.10  N=9 | 0.50±0.10  N=10 |
| **5HIAA**  Genotype effect: F_(1,33)_=0.000805; p=0.09775  Sex effect:  F_(1,33)_=0.000609; p=0.9383  Interaction genotype*sex:  F_(1,33)_=0.1022; p=0.7512 | 0.45±0.11  N=8 | 0.47±0.08  N=10 | 0.47±0.08  N=9 | 0.44±0.07  N=10 |
| **HVA**  Genotype effect: F_(1,30)_=1.41814; p=0.23371  Sex effect:  F_(1,30)_=0.6115; p=0.43422  Interaction genotype*sex:  F_(1,30)_=0.09702; p=0.75543 | 0.25±0.05  N=8 | 0.26±0.06  N=10 | 0.38±0.09  N=6 | 0.28±0.05  N=10 |
| **3MT**  Genotype effect: F_(1,31)_=0.125; p=0.72371  Sex effect:  F_(1,31)_=0.1021; p=0.74934  Interaction genotype*sex:  F_(1,31)_=5.8202; p=0.01584 | 0.54±0.09  N=9 | 1.03±0.22  N=9 | **1.14±0.28**  N=8 | 0.66±0.16  N=9 |
| **5HIAA/5-HT ratio**  Genotype effect: F_(1,33)_=0.01823; p=0.8926  Sex effect:  F_(1,33)_=0.33044; p=0.5654  Interaction genotype*sex:  F_(1,33)_=0.36514; p=0.54567 | 0.61±0.06  N=8 | 0.63±0.06  N=10 | 2.66±1.68  N=9 | 1.82±1.26  N=10 |
| **DOPAC/DA ratio**  Genotype effect: F_(1,34)_=0.000213; p=0.98835  Sex effect:  F_(1,34)_=0.167521; p=0.68232  Interaction genotype*sex:  F_(1,34)_=0.017511; p=0.89472 | 0.20±0.03  N=8 | 0.22±0.04  N=10 | 1.37±1.01  N=9 | 0.73±0.50  N=10 |
| **HVA/DA ratio**  Genotype effect: F_(1,30)_=0.24661; p=0.61947  Sex effect:  F_(1,30)_=0.04389; p=0.83405  Interaction genotype*sex:  F_(1,30)_=0.71597; p=0.39747 | 0.12±0.02  N=8 | 0.13±0.02  N=10 | 1.49±1.10  N=6 | 0.35±0.24  N=10 |
| **3MT/DA**  Genotype effect: F_(1,31)_=0.2832; p=0.59459  Sex effect:  F_(1,31)_=0.0218; p=0.88265  Interaction genotype*sex:  F_(1,31)_=5.5623; p=0.01835 | 0.30±0.06  N=9 | 0.66±0.22  N=9 | 4.02±2.97  N=8 | 1.13±0.89  N=9 |
